# Interplay Among the Oral Microbiome, Oral Cavity Conditions, the Host Immune Response, Diabetes Mellitus, and Its Associated-Risk Factors—An Overview

**DOI:** 10.3389/froh.2021.697428

**Published:** 2021-09-09

**Authors:** Thais de Cássia Negrini, Iracilda Zeppone Carlos, Cristiane Duque, Karina Sampaio Caiaffa, Rodrigo Alex Arthur

**Affiliations:** ^1^Department of Clinical Analysis, School of Pharmaceutical Sciences, São Paulo State University, Araraquara, Brazil; ^2^Department of Restorative and Preventive Dentistry, Araçatuba Dental School, São Paulo State University, Araçatuba, Brazil; ^3^Department of Preventive and Community Dentistry, Dental School, Federal University of Rio Grande do Sul, Porto Alegre, Brazil

**Keywords:** diabetes mellitus, hyperglycemia, oral cavity, oral microbiome, immune response, obesity, hypertension

## Abstract

This comprehensive review of the literature aimed to investigate the interplay between the oral microbiome, oral cavity conditions, and host immune response in Diabetes mellitus (DM). Moreover, this review also aimed to investigate how DM related risk factors, such as advanced age, hyperglycemia, hyperlipidemia, obesity, hypertension and polycystic ovary syndrome (PCOS), act in promoting or modifying specific mechanisms that could potentially perpetuate both altered systemic and oral conditions. We found that poorly controlled glycemic index may exert a negative effect on the immune system of affected individuals, leading to a deficient immune response or to an exacerbation of the inflammatory response exacerbating DM-related complications. Hyperglycemia induces alterations in the oral microbiome since poor glycemic control is associated with increased levels and frequencies of periodontal pathogens in the subgingival biofilm of individuals with DM. A bidirectional relationship between periodontal diseases and DM has been suggested: DM patients may have an exaggerated inflammatory response, poor repair and bone resorption that aggravates periodontal disease whereas the increased levels of systemic pro-inflammatory mediators found in individuals affected with periodontal disease exacerbates insulin resistance. SARS-CoV-2 infection may represent an aggravating factor for individuals with DM. Individuals with DM tend to have low salivary flow and a high prevalence of xerostomia, but the association between prevalence/experience of dental caries and DM is still unclear. DM has also been associated to the development of lesions in the oral mucosa, especially potentially malignant ones and those associated with fungal infections. Obesity plays an important role in the induction and progression of DM. Co-affected obese and DM individuals tend to present worse oral health conditions. A decrease in HDL and, an increase in triglycerides bloodstream levels seem to be associated with an increase on the load of periodontopathogens on oral cavity. Moreover, DM may increase the likelihood of halitosis. Prevalence of impaired taste perception and impaired smell recognition tend to be greater in DM patients. An important interplay among oral cavity microbiome, DM, obesity and hypertension has been proposed as the reduction of nitrate into nitrite, in addition to contribute to lowering of blood pressure, reduces oxidative stress and increases insulin secretion, being these effects desirable for the control of obesity and DM. Women with PCOS tend to present a distinct oral microbial composition and an elevated systemic response to selective members of this microbial community, but the association between oral microbiome, PCOS are DM is still unknown. The results of the studies presented in this review suggest the interplay among the oral microbiome, oral cavity conditions, host immune response and DM and some of the DM associated risk factors exist. DM individuals need to be encouraged and motivated for an adequate oral health care. In addition, these results show the importance of adopting multidisciplinary management of DM and of strengthening physicians-dentists relationship focusing on both systemic and on oral cavity conditions of DM patients.

## Introduction

The mouth harbors a highly heterogeneous ecosystem. The microorganisms found in the human oral cavity have been referred to as the oral microflora, oral microbiota, or more recently as the oral microbiome. As cited by Dewhirst et al. [1], the term microbiome was coined by Joshua Lederberg “to signify the ecological community of commensal, symbiotic, and pathogenic microorganisms that literally share our body space and have been all but ignored as determinants of health and disease” [1, 2]. The oral cavity includes several distinct microbial habitats, such as teeth, gingival sulcus, attached gingiva, tongue, cheek, lip, hard palate, and soft palate [1]. Dental materials used for rehabilitation (restorations, prostheses, and implants) are also habitats for microorganisms and allow the formation of extensive and long-lasting polymicrobial biofilms [3, 4]. Proteins from saliva and gingival crevicular fluid (either directly or indirectly) regulate microbial establishment and growth in the oral cavity, either by providing molecules for microbial attachment, by acting as nutrients for microbial growth, or by exerting an antimicrobial effect [4, 5].

The oral microbiome is composed of more than 700 microbial species, of which 57% are officially named, 13% are unnamed but cultivated, and around 30% are represented by uncultivated species [6]. Relative stability of the bacterial community has been reported over time within the same oral environment [7, 8]. The persistence of this commensal microbiota is extremely important to promote the development of proper tissue structure and function and to prevent pathogenic microorganism colonization. Therefore, the commensal microbiota is responsible for maintaining normal physiology and health in humans [9, 10].

Generally, a high abundance of *Streptococcus* ssp., *Gemella* ssp., *Rothia* ssp., *Neisseria* spp., *Haemophilus* spp., *Prevotella* spp., and *Veillonela* spp. is found in the oral cavity of healthy individuals [11–15]. Several fungal genera are also found in the oral cavity, with predominance of the genera *Candida* spp., followed by *Aspergillus* spp., *Penicillium* spp., *Schizophyllum* spp., *Rhodotorula* spp., and *Gibberella* spp. [16]. Moreover, virus, phages, protozoa, and archaea are also part of the oral microbiome [17, 18]. The composition of the oral microbiome can be disturbed by dietary habits, tobacco use or alcohol consumption, stress, hormonal oscillation during puberty and pregnancy, poor oral hygiene, and systemic diseases [4, 19], which may lead to imbalance in host-microbiome interactions. This imbalance induced by microbial shifts (also denominated dysbiosis) leads to a health-to-disease state transition. There is increasing scientific evidence supporting that many systemic diseases, such as metabolic disorders, cardiovascular diseases, and cancer, are associated with disturbances in the oral microbiome [20]. One of the possible explanations for this association is that an altered oral microbiome can trigger both local and systemic immune-inflammatory processes that are directly or indirectly related to some of these systemic diseases [21].

Diabetes mellitus (DM) is a metabolic disorder associated with chronic hyperglycemia that occurs as a result of impaired insulin secretion and/or insulin resistance. Insulin is a hormone produced by β cells of the pancreatic islets of Langerhans and its primary function is to maintain the homeostasis of blood sugar levels. This hormone is secreted in response to the increase in circulating levels of glucose and amino acids after a meal. In order to maintain blood sugar levels, this hormone reduces hepatic glucose production (reducing gluconeogenesis and glycogenolysis) and increases the rate of glucose uptake, especially in striated muscle and adipose tissue. In addition, insulin also affects lipid metabolism, increasing lipid synthesis in the liver and fat cells and attenuating the release of fatty acids from triglycerides in adipose tissue and muscles. Insulin resistance occurs when normal circulating hormone concentrations are insufficient to adequately regulate these processes [22].

Although studies on insulin action have traditionally focused on the peripheral tissue domain, more and more preclinical and clinical studies are being reported focusing on the effects of insulin resistance on the central nervous system [23], since an effect of insulin resistance in the brain was also observed in obese, DM, older, and dementia individuals [24]. Insulin receptors are present throughout the brain, therefore this hormone plays important roles not only in the metabolism of the whole body, but also in brain functioning, being, therefore, involved in neuropathological processes [25]. This occurs because insulin is able to cross the blood-brain barrier. Once binded to its receptor, insulin activates signaling cascades that regulate the systemic breakdown of nutrients. As a result, insulin controls appetite, adipose tissue lipolysis, hepatic triglyceride secretion, and branched-chain amino acid metabolism, protecting the body from ectopic lipid accumulation and lipotoxicity. Overnutrition rapidly induces insulin resistance in the brain, even before peripheral insulin signaling is impaired, implicating insulin resistance in the brain as a major culprit in diabetes [26]. In addition, insulin influences brain bioenergetics, increasing synaptic viability and the turnover of neurotransmitters such as dopamine. Furthermore, it also influences the clearance of β amyloid peptide and the phosphorylation of tau protein, which are determining factors in the pathophysiology of Alzheimer's disease. This hormone also modulates vascular function through effects on vasoreactivity, lipid metabolism, and inflammation. Therefore, through these multiple pathways, insulin dysregulation can contribute to neurodegeneration [27].

According to the American Diabetes Association [28], DM can be classified into four categories: type 1 (DM1), type 2 (DM2), gestational DM (DMG), and DM associated with other causes [28]. The understanding of DM1 has been changing over the years [29]. Previously, DM1 was considered a chronic and single autoimmune disease, classically characterized by a deficiency in insulin production due to a T-cell mediated attack on insulin-producing pancreatic β-cells, which resulted in hyperglycemia. This cell destruction is traceable by the detection of a variety of autoantibodies. However, there are patients with DM1 who are negative for the detection of these autoantibodies, which shows that DM1 may also be classified as non-immune-mediated idiopathic DM1 [30]. More current concepts show that DM1 is the result of a network of interactions between environmental factors and patient-related factors, such as microbiome, genome, metabolism, and the immune system, with the contribution of each of these factors being variable among affected individuals [29]. DM2 is a chronic disease represented by a complex set of metabolic alterations. Although the specific etiology of this DM type is not completely known, it is clear that it is not related to the autoimmune destruction of β-pancreatic cells [31]. DM2 is a pandemic disease worldwide, accounting for 90–95% of all diagnosed diabetes cases [28]. One of the characteristics of DM2 is the presence of peripheral insulin resistance in tissues such as skeletal muscle, adipose tissue, and liver. This resistance causes an increase in insulin production, which in turn leads to an increase in β-cell mass and more insulin secretion in order to maintain normoglycemia. This compensatory response results in hypersecretion of insulin, leading to the development of hyperinsulinemia. In a vicious cycle, hyperinsulinemia exacerbates metabolic dysregulation in both obesity and DM2 patients. Insulin resistance and hyperinsulinemia precede the development of hyperglycemia that occurs when β-cells fail to compensate for peripheral insulin resistance. In the development of DM2, several factors such as the presence of cytokines, free fatty acids, and hyperglycemia have been suggested as mediators of β-cell decompensation [32]. Furthermore, decreased levels and low activity of metabolic enzymes involved in glucose metabolism, especially mitochondrial enzymes, were also associated with the β-cell decompensation process in DM2 [32, 33]. Advanced age, obesity, dyslipidemia, hypertension, hyperglycemia, polycystic ovary syndrome, and immune dysfunction are among the most common risk factors associated with DM2 [34, 35]. DMG is the most common medical disorder in pregnancy, generally diagnosed between the second and third trimester of pregnancy (this condition is absent before pregnancy) [36]. Finally, monogenic diabetes syndromes (such as neonatal diabetes and juvenile diabetes), exocrine pancreatic diseases (such as cystic fibrosis and pancreatitis), or drug-induced diabetes (such as the use of glucocorticoids) are other causes of DM [28].

Chronic hyperglycemia as a result of poorly controlled diabetes in patients may increase the risk of developing several complications in the eyes (retinopathy with potential loss of vision), kidneys (nephropathy that can cause renal failure), nerves (peripheral neuropathy with risk of ulcers in the feet), heart (atherosclerotic cardiovascular and peripheral arterial diseases), and blood vessels (cerebrovascular disease) [37]. According to the World Health Organization, approximately 422 million people worldwide have DM and 1.6 million deaths are directly attributed to diabetes each year [38]. In 2045, it is expected that the number of diabetic patients will exceed 600 million people [39]. The multifactorial nature of this disease makes patient clinical management, which includes drug therapy throughout life and lifestyle modification, extremely challenging [40].

A recent systematic review found that diabetic patients have inadequate or limited knowledge and attitudes about their oral health and do not visit their dentists very often. Moreover, uncontrolled diabetic patients are at a greater risk of presenting oral health problems. Provision of oral health education by diabetes care providers and referral to dentists were associated with improved oral health behaviors among patients [41]. This means that comprehensive management of these patients, including the management of both systemic and oral health, is needed. Moreover, a role of the microbial communities as potential contributors to DM state has even been envisaged [39], although the mechanisms involved in the interplay among the oral microbiome, oral cavity conditions, immune response, DM, and its associated risk factors are NOT clear enough.

Therefore, this comprehensive review of the literature aimed to investigate the interplay between the oral microbiome, oral cavity conditions, and host immune response in DM. Moreover, this review also aimed to investigate how DM related risk factors, such as advanced age, hyperglycemia, hyperlipidemia, obesity, hypertension, and polycystic ovary syndrome (PCOS), act in promoting or modifying specific mechanisms that could potentially perpetuate both altered systemic and oral conditions.

## Main Findings and Discussion

The following section is focused on the systemic- and/or oral cavity-related features that are modified in DM patients and potentially related to the disease. Additional information about the general aspects of the oral cavity and the general features of the immune response can be found in the Supplementary Material.

### Immune Response in DM: Potential Factors Associated With Increased Susceptibility to Infections

Several aspects of the immune system can be altered depending on the DM-associated risk factors. This topic covers the factors that lead to increased susceptibility to infections in diabetics.

Immune dysfunction plays a key role in the pathogenesis of DM-associated infections. DM may exert a negative effect on the immune system, leading to a deficient immune response in diabetic individuals [42]. Combined with metabolic imbalance, impairment of the immune response is primarily responsible for the increased susceptibility and prevalence of infections in these individuals [35, 43], with more severe infectious courses and greater morbidity and mortality compared to non-diabetic individuals [44]. A systematic review carried out by Jafar et al. [45] evaluating the effects of hyperglycemia on the innate immune system showed that hyperglycemia activates protein kinase C, which causes the inhibition of neutrophil migration, phagocytosis, and superoxide production, leading to decreased microbial death. In addition, high glucose blood levels inhibit functional activity of neutrophils, decreasing the formation of their extracellular traps [45].

Another reason for the increased susceptibility to infection is possible impaired activation of TLR (Toll-like receptor) in DM individuals [46]. Neutrophils from diabetic rats (DM2) were shown to lack TLR-4 activation in the presence of microbial lipopolysaccharide (LPS), leading to impaired cytokine production [46]. Stegenga et al. [47] verified the effects of hyperglycemia and hyperinsulinemia on immune and hemostatic responses during systemic inflammation. For this, a human endotoxemia model [48] was used to induce an inflammatory and pro-coagulant response in healthy individuals who were challenged with intravenous LPS in the presence of one of the following factors: (1) normal glucose and insulin concentrations, (2) hyperglycemia with low insulin levels, (3) hyperinsulinemia with normal glucose levels, or (4) hyperglycemia and hyperinsulinemia combined. The results showed that hyperglycemia impaired neutrophil degranulation and potentiated coagulation, while hyperinsulinemia inhibited fibrinolysis, suggesting that patients with DM2 may be especially vulnerable to prothrombotic events during inflammatory states. In addition, hyperglycemia can also alter the complement structure, inhibiting the opsonization of microorganisms and decreasing phagocytosis processes [45]. This decreased phagocytosis seems to favor the microbial growth of some microorganisms that also become more virulent, such as *Candida albicans* which takes advantage of glucose-rich environments that increase fungal adherence to epithelial and endothelial cells in patients with diabetes [49]. Consequently, poor glycemic control is associated with an increased risk of hospitalizations for serious long-term infections, particularly bone and joint infections, endocarditis, tuberculosis, and sepsis [50]. Furthermore, DM individuals are more prone to new or recurrent infections, for example, urinary tract infection, respiratory tract infection, skin and soft tissue infection (including diabetic foot), osteomyelitis, and peritonitis. In addition, some uncommon life-threatening diseases (such as necrotizing soft tissue infection, emphysematous pyelonephritis, emphysematous cholecystitis, malignant otitis, and perioperative infection) are more frequent in DM individuals [39, 51]. Evidence suggests that the reduction in blood glucose levels allows improvement in neutrophil functional activity [52].

On the contrary, although studies show that diabetes predisposes the host to worse infection outcomes due to *suppression* of the immune system, other studies show that the worst outcomes of an infectious process in diabetic patients are caused by *overstimulation* of the immune system. In this intricate setting, the transmembrane receptor RAGE, TLRs, and their activation axes appear to have particular importance in orchestrating a multitude of pro-inflammatory cellular responses that lead to many of the complications and damages reported in patients with DM.

RAGE was first described as a receptor for advanced glycation end products (AGEs). It is expressed in different cell types, including macrophages, and the effect of the AGE-RAGE axis causes the expression of inflammatory mediators by these cells. AGEs are a heterogeneous group of non-enzymatically produced molecules from the interaction between reducing sugars or reactive oxoaldehyde and proteins, nucleic acids, and lipids [53]. These molecules are normally synthesized during metabolism, but they can also be uptaken from the diet: sugar-rich foods are considered the main exogenous source of AGEs, exerting a significant influence on the development of several pathological complications in diabetic patients [54]. AGEs interfere with the normal functioning of almost all body organs by exerting multiple actions such as apoptosis, inflammation, protein dysfunction, mitochondrial dysfunction, and oxidative stress [55]. Studies suggest that a diet low in AGEs has beneficial effects on insulin resistance and on fasting insulin, total cholesterol, and LDL levels [56]. Insulin glycation reduces its biological activity by decreasing its affinity for the insulin receptor. Moreover, glycated insulin can act as a ligand for RAGE, activating both oxidative stress and inflammatory pathways that lead to insulin resistance [57]. Since AGE levels are markedly increased in diabetics, it has been suggested that they can be used as clinical biomarkers for the presence of complications in these patients [58–60].

In addition to being a receptor for AGEs, RAGE has also been recognized as a multi-linker receptor, including for high mobility group box 1 (HMGB1) whose activation pathway can also contribute to the production of pro-inflammatory mediators [61]. HMGB1 is an abundant nuclear and cytoplasmic protein present in mammalian cells. It is a DNA-binding protein involved in maintaining the structure of the nucleosome and regulating genetic transcription. In addition to these intracellular functions, HMGB1 is also released to the extracellular environment by activated innate immune cells, where it acts as an alarmin for the immune system, causing systemic inflammatory responses [62]. In addition to the interaction with RAGE, several members of the TLR family, including TLR2, 4, 5, and 9, are also able to interact with HMGB1 [63–66]. HMGB1 therefore acts by activating RAGE and TLRs, contributing to the production of pro-inflammatory mediators associated with DM [67].

As mentioned above, the worst infection outcomes in diabetics are caused by overstimulation of the immune system, by the activation of RAGE, and by elevated levels of AGEs. In this line of investigation, Nielsen et al. [68] showed that diabetic mice were hypersusceptible to bacteremia caused by Gram-negative bacteria. The authors noticed that the susceptibility was not related to the bacterial load *per se*, but because diabetic mice exhibited markedly increased levels of pro-inflammatory cytokines in response to these bacteria. The authors observed simultaneous activation of TLR4 and RAGE, both being receptors related to signaling of a common downstream messenger, MyD88, triggering hyperinflammation. Therefore, the severe infection in diabetics is also related to additional inflammatory mediator production through the dual activation of MyD88. Moreover, the authors also showed that inactivation of the RAGE axis seems to protect diabetic animals, but not non-diabetic animals, from infection. This could be explained by the interruption of RAGE, which is upregulated by elevated levels of AGEs. Thus, instead of promoting immunosuppression, diabetes stimulated lethal hyperinflammation in response to infection through RAGE signaling [68].

It has been reported that an excess of glucose in the systemic circulation can cause an increase in the generation of reactive oxygen species (ROS), IL-1, IL-6, and TGF-β. Activation of the AGE/RAGE axis due to high glucose blood levels also initiates the production of glutathione (GSH) which has the function of absorbing and detoxifying ROS, preventing cell damage. The oxidized form of glutathione (GSSG) can be recycled using the enzyme glutathione reductase (GSR) if the body has sufficient quantities of the reduced form of adenine dinucleotide nicotinamide phosphate (NADPH). However, in diabetic patients, excess glucose is not oxidized but is redirected to a polyol pathway where NADPH is heavily used. This leads to a general decrease in GSH and an increase in ROS levels, which alter the cytokine production profile and, consequently, the immune response in these patients [69, 70]. It has been shown that this change in cytokine production increases the susceptibility to *Mycobacterium tuberculosis* infection [71, 72]. Conversely, the increase in GSH positively regulates the production of IFN-γ and IL-12, favoring the immune response in DM2 against infection by this microorganism [69, 70]. In this way, DM increases the risk of developing tuberculosis by about three times, doubles the risk of death during treatment of the disease, and can also increase the risk of infection by latent *M. tuberculosis* in diabetic patients [73].

Therefore, hyperglycemia and the immune system can differentially impact the production of inflammatory mediators during microbial infection in diabetic individuals. These changes may not only actively participate in the inflammatory process of DM, accelerating the complications associated with the disease, but may also interfere with the host's defense against microbial infections [74, 75].

### Main Oral Cavity Manifestations in DM Affected Individuals

The following section is focused on oral cavity manifestations directly or indirectly associated with DM. More information about general aspects of hyposalivation and/or xerostomia, dental caries, periodontal diseases, halitosis and taste perception/ smell recognition are described in the Supplementary Material.

#### Xerostomia and Hyposalivation

Saliva is a complex fluid produced by the salivary glands. It has multiple functions which are essential for maintaining oral and general health. This fluid has a wide variety of constituents and biological properties, which are important for the protection of teeth and the oropharyngeal mucosa, as well as facilitating speech, and saliva is essential for the processes of chewing and swallowing. In addition to these functions, and due to its antimicrobial components, saliva also regulates the composition of the oral microbiome [76]. Hyposalivation (a common signal of salivary gland hypofunction) is diagnosed when the salivary flow rate is below the normal expected range of 0.7–2.0 mL/min for stimulated- and of 0.2–0.4 mL/min for unstimulated collected saliva. On the other hand, xerostomia is defined as a subjective and self-reported complaint of dry mouth. It is important to mention that xerostomia may or may not be associated with hyposalivation.

Diabetic patients present hyposalivation and xerostomia. A systematic review carried out by López-Pintor et al. [77] showed lower salivary flow rates and a higher prevalence of xerostomia in diabetic patients (from 12.5 to 53.5%) compared with healthy individuals (from 0 to 30%). In a cross-sectional study, Pappa et al. [78] investigated how the level of diabetic control affects salivary function, the prevalence of dry mouth, and the experience of caries in children and adolescents with DM1. One hundred-fifty children and adolescents (10–18 years) were examined and allocated into 3 groups: 50 poorly- controlled diabetic patients (HbA1c ≥ 7.5%), 50 well-controlled diabetic patients (HbA1c <7.5%), and 50 healthy controls. All individuals were examined for dental caries and salivary factors. Assessments of salivary characteristics included self-reported xerostomia, quantification of the stimulated and resting total saliva flow rates, pH values, buffer capacity, and saliva viscosity. Caries experience was recorded using the DMFT index. Greater caries experience, a higher prevalence of dry mouth, and a decrease in the unstimulated salivary flow rate were found in poorly controlled diabetic patients [78]. Sialosis is a disease characterized by an increase in the volume of salivary glands, especially in the parotids. This disease is characterized by adipose infiltration into the glandular parenchyma, leading to gland swelling and loss of salivary production. This condition is commonly found in diabetic patients, being one of the causes of xerostomia [79, 80]. Therefore, this evidence suggests that salivary flow rate tends to be lower, whereas the prevalence of xerostomia tends to be higher in diabetic patients.

#### Dental Caries

Evidence suggests that the number of total streptococci and lactobacilli in supragingival biofilm and the incidence of active dental carious lesions were higher in DM than in healthy individuals [81]. More recently, studies based on 16S rRNA gene sequencing identified a significant difference in the salivary oral microbiome of DM2 patients compared to healthy individuals, including an increased ratio of Firmicutes/Bacteroidetes and high abundance of *Haemophilus* spp. in DM patients [82]. *Actinomyces* spp. and *Selenomonas* spp. were also more abundant in the saliva of older DM2 patients than in healthy individuals [83]. Goodson et al. [84] showed that the counts of several bacteria were smaller in individuals who presented high salivary glucose levels compared with healthy individuals as follows: *Prevotella melaninogenica, Prevotella nigrescens, Prevotella intermedia, Aggregatibacter actinomycetemcomitans, Propionibacterium acnes, Actinomyces naeslundii, Actinomyces odontolyticus, Actinomyces gerencseriae, Actinomyces viscosus, Actinomyces israelli, Gemella morbillorum, Streptococcus salivarius, Streptococcus oralis, Streptococcus anginosus, Streptococcus intermedius, Streptococcus contellatus, Streptococcus gordonii, Streptococcus sanguinis, Streptococcus mitis, Streptococcus mutans, Eikenella corrodens, Veillonella parvula, Fusobacterium nucletum, Tannerella forsythia, Selenomonas noxia, Porphyromonas gingivalis, Capnocytophaga gingivalis, Capnocytophaga ochraceae, Campylobacter rectus, Campylobacter showae, Campylobacter gracilis, Lachnoanaerobaculum saburreum*, and *Treponema denticola*. However, *Parvimonas micra* showed an opposite trend. It was hypothesized that the salivary acidification induced by hyperglycemia might contribute to the observed changes. These authors also showed that patients with high salivary glucose levels had a greater caries experience than those with low glucose levels [84].

Conflicting data about the association between DM and dental caries are found in systematic reviews. Ismail et al. [85] emphasize that from 15 included studies, 8 showed a greater caries experience in DM1 children/adolescents, whereas in 7 studies this difference in caries experience was not observed between DM and healthy patients. Distinct levels of glycemic control of the studied individuals might contribute to this lack of association. On the other hand, other systematic reviews and meta-analysis showed a greater dental caries experience in DM individuals. DM1 patients showed a significantly higher caries experience in permanent teeth than healthy individuals [86] but no significant differences were found either between DM2 patients and healthy individuals, between well-controlled and poorly controlled DM patients, or regarding diabetes duration and caries experience [86], although several individual studies did find correlations between metabolic control and caries experience [87–89]. It was discussed that high heterogeneity regarding age cut-offs of participants and the level of glycosylated hemoglobin reported by the included studies impacted the significance of the meta-analysis [86]. Moreover, adults with DM and with uncontrolled glycemic levels also showed a greater caries experience and greater caries prevalence than healthy individuals [90]. Additionally, DM2 patients were three times more likely to have root caries than healthy individuals [90]. The presence of dental caries might be an oral sign to be considered as an indicative of poor glycemic control in DM patients.

#### Periodontal Diseases

Periodontal disease is a well-known oral condition, frequently associated with the pathogenesis of several systemic diseases, including DM [91]. Scientific evidence has appointed a bidirectional interrelationship between periodontal diseases and DM [92–95], as a consequence of the inflammatory characteristic of both diseases. Patients with periodontitis have a 27–53% higher risk of developing DM than healthy individuals, suggesting that periodontitis is an aggravating factor in the incidence of DM [96]. Elevated pro-inflammatory mediators in the periodontal tissue of patients with poorly controlled DM suggest a biological pathway that can aggravate periodontal disease [97]. Possible mechanisms of how DM affects periodontitis have been proposed, such as the neutrophil dysfunction with higher levels of cytokines and less effective bacterial killing, abnormal collagen metabolism, and increased interaction of AGE-RAGE, which lead to an exaggerated inflammatory response and impaired repair and bone reabsorption [93–95]. The effect of periodontal disease on DM is potentially explained by the resulting increase in the levels of systemic pro-inflammatory mediators, which exacerbates insulin resistance [93]. Healthy individuals with periodontitis have low glycemic control and an increased risk of developing DM. In addition, DM individuals present worsening glycemic control if they are also affected by periodontitis and a higher prevalence of complications related to DM [98]. It is important to point out that although the authors concluded that periodontal disease impacts on the control, incidence, and complications of DM, they also acknowledged that further evidence is needed, considering the high heterogeneity and risk of bias of the assessed studies [98].

Studies in humans and mice support the concept that Th1 cells and their cytokines characterize early stages of periodontal diseases, while Th2 cells are associated with disease progression. However, evidence shows that periodontal disease is not adequately described only by the dichotomy between Th1 and Th2, since Th17 is also involved in host-pathogen interactions in the periodontium [99]. The deleterious effect caused by DM on bone increases the risk of bone fracture and formation of osteolytic lesions, such as those that occur in periodontitis. One of the cytokines involved in this process is IL-17 [100]. Huang et al. [101] discuss that there is intensification of IL-17 production in DM individuals, causing an increase in gingival inflammation and induces changes in the composition of the oral microbiota, which becomes more pathogenic [101]. In addition, IL-17 also induces osteoclastogenesis, which promotes bone resorption of periodontal tissue, leading to tooth loss [101, 102]. It was demonstrated, though, that by locally treating periodontal tissue in mice with antibodies against IL-17, bone loss was interrupted, as well as which, a reduction in the total bacterial load in periodontal tissue was observed [100]. Osteoclast maturation, and bone modeling and remodeling are controlled by the signaling system composed of the receptor activator of nuclear factor kappa-B (NF-kB) (RANK), RANK ligand (RANKL), and osteoprotegerin (OPG). RANK binds to its receptor RANKL and increases osteoclast differentiation and maturation and OPG binds to RANKL to inhibit these processes. Hyperglycemia and increased inflammation caused by DM lead to an increase in RANKL and a decrease in OPG, which contribute to greater bone resorption [103, 104]. Both diet and exercise can promote bone health through RANKL/RANK/OPG pathway regulation [105–108]. Diets with soy-containing isoflavones and zinc supplementation significantly increase OPG protein levels and the OPG/RANKL ratio in DM2 [107, 108]. Natural β-glucans are the main structural components of fungi, plants, cereal grains, and some bacterial cell walls and have been shown to reduce bone loss, blood glucose, cholesterol, and triacylglycerol levels in models of periodontal diseases and DM [109, 110]. They also reduced the expression of the receptor activator of NF-kB and increased OPG expression in animals with DM and periodontal disease [109, 110]. Physical exercise has been shown to improve glycemic control and the systemic inflammatory profile, reducing TNF-α (which is involved in the inflammatory process and in insulin resistance, impairing bone remodeling, and accelerating the destruction of periodontal tissues), increasing IL-10 (which exerts an inhibitory effect on TNF-α elevation), and increasing the IL10/TNF-α ratio. The TNF-α/IL-10 ratio is an indicator of the pro-inflammatory status, and the higher this ratio, the greater the pro-inflammatory profile (the IL-10/TNF-α ratio indicates the opposite). Thus, there is attenuation of alveolar bone loss in cases where there is DM with periodontal disease [111–113].

Some investigations have been able to identify changes in the composition of the oral microbiota within the bidirectional relationship between DM and periodontitis [114, 115]. Shi et al. [114] characterized the subgingival microbiome of DM2 and non-diabetic individuals, in either the healthy periodontal state, periodontitis state, or after periodontal treatment [114]. DM2 patients were more susceptible to shifts in the subgingival microbiome toward dysbiosis and in developing periodontitis. The authors noted a high correlation of pathogenic species not only in the periodontitis condition, but also in the healthy state in DM2, suggesting an elevated risk of progression to periodontitis. Moreover, abundance of Fusobacteria and Actinobacteria were greater in the subgingival biofilm of DM individuals presenting periodontal disease, whereas Proteobacteria was less abundant in that condition [115]. In contrast, no differences in Actinobacteria or Bacteroidetes abundances were found between DM patients presenting or not a probing depth (PD) ≥4 mm. The presence of Actinobacteria significantly increased the odds for DM by 10% in subjects with gingival bleeding, while the presence of Fusobacteria increased the odds by 14%, yet, among subjects with PD ≥4 mm, Fusobacteria decreased the odds of DM by 47% [115].

In addition, by comparing the salivary microbiota of impaired glucose tolerance, DM2, and normoglycemic patients, Saeb et al. [116] showed a reduction in the microbial diversity in DM2 and prediabetes patients in comparison with normoglycemic patients. Similarly, Farina et al. [117] showed that the subgingival microbiome of DM2 and/or periodontitis patients presented decreased richness and diversity in relation to healthy individuals.

By reducing the intraoral microbial load and the level of periodontal inflammation, periodontal therapy may have a significant impact on the systemic inflammatory state. Periodontal therapeutic interventions have been associated with better glycemic control in DM patients [118]. The study of D'Aiuto et al. [119] showed that effective periodontal treatment reduces systemic inflammatory markers and favorably influences the metabolic status of DM2 patients, who showed reduced glucose and HbA1c plasma levels, as well as improving the vascular and renal functions of these patients [119, 120]. A systematic review and meta-analysis of randomized controlled trials showed that periodontal therapy significantly reduced the level of HbA1c after 3 and 6 months. Periodontal therapy seems to contribute significantly to glycemic control in DM2 patients, especially in patients with higher baseline levels of HbA1c [121].

The placement of dental implants is one of the treatments used to replace missing teeth. Dental implants have become an integral treatment in dentistry for both total or partial edentulism. Jiang et al. [122] carried out a systematic review to explore a possible association between DM and dental implant complications. The authors reported that marginal bone loss, probing depth, and bleeding around dental implants were greater in DM patients than in healthy individuals. The authors also showed that bleeding around the implants increased as the level of HbA1c increased [122].

#### Oral Mucosal Lesions

Lesions that affect the oral mucosal surfaces can manifest in several ways and comprise a wide spectrum of benign or malignant lesions. Vasconcelos et al. [123] evaluated the prevalence of some mucosal manifestations in the oral cavity of DM and found that most DM patients had at least one type of the following manifestations: lingual varicosity, erythematous candidiasis, angular cheilitis, traumatic ulcer, fissured tongue, gingival hyperplasia, mucocele, petechiae, hyperkeratosis, and atrophy of lingual papillae. Tongue varicose veins (36.6%) and candidiasis (27.02%) were the most prevalent. These lesions may also be associated with the fact that these conditions are commonly found in older patients and are also associated with prolonged use of prostheses [123]. Bastos et al. [124] and González-Serrano et al. [125] found a higher prevalence of alterations in the normal aspect of oral mucosa in DM patients, especially with a significant difference for potentially malignant diseases and fungal infections. Most of the lesions seem to be found on the tongue or associated with denture use [125]. An increased proportion of yeasts have been found associated with mucosal lesions in DM patients, with *C. albicans* being the most prevalent, followed by *Candida tropicalis* and *Candida krusei* [126]. These authors also suggested the presence of DM as a risk factor for changes in the normal aspect of oral mucosa. Moreover, it has been discussed that the response to several cancer therapies may be altered due to the interaction between the microbiome and the human body [127]. Ramos-Garcia et al. [128] found that DM patients with oral cancer present higher mortality when compared to non-diabetic patients with oral cancer. Additionally, DM patients have a higher chance of developing tumors when compared to non-diabetic individuals. Considering that there is growing evidence associating specific oral taxon and the onset and progression of oral cancer, it is tempting to consider that DM-associated changes in the oral microbiome might be linked to a poorer cancer prognosis.

In addition to the above discussed oral manifestations, comparisons between non-diabetic and DM affected individuals regarding halitosis, taste perception, and smell recognition are presented in the Supplementary Material.

#### COVID-19, the Oral Cavity, and DM

Coronavirus disease 2019 (COVID-19), caused by the SARS-CoV-2 virus, which suddenly appeared in December 2019, is still affecting people worldwide and putting health systems and the global economy under stress. By January 2021, there were approximately 98.0 million confirmed cases and 2.0 million confirmed deaths [129]. SARS-CoV-2 is usually transmitted by inhalation or contact with infected droplets from a contaminated individual. The incubation period for the virus is approximately 2–14 days. Therefore, during this period, virus particles are present in the secretions of infected people and it has been proven that asymptomatic individuals can also transmit the virus to other people [130]. In symptomatic patients with COVID-19, the course of the disease can be didactically divided into four phases. Phase 1: Viral entry into host cells. This phase begins when the individual becomes symptomatic and the most common clinical manifestations are fever, dry cough, loss of taste perception and smell recognition, and general malaise. For most individuals, the disease is limited to this phase. The transition from Phase 1 to Phase 2 is characterized by migration of immune cells to the lungs. Phase 2 is the lung stage of the disease, that is, when individuals develop pulmonary inflammation and pneumonia. In this phase, the response of interferon to the virus is impaired and there is a storm of cytokine production. Based on the presence or absence of hypoxia, this phase can be subdivided into IIa (without hypoxia) or IIb (with hypoxia). At this stage, most individuals require hospitalization and those with prolonged hypoxia need mechanical ventilation. In Phase 3, patients develop acute respiratory syndrome and extrapulmonary systemic hyperinflammation syndrome, shock, vasoplegia, respiratory failure, cardiopulmonary collapse, myocarditis, and acute kidney injury, with a worse prognosis and increased mortality. Phase 4 is characterized by the recovery and survival phase [131, 132].

The oral cavity is an important reservoir of SARS-CoV-2. The virus is found in nasopharyngeal secretions and its viral load is consistently high in saliva, especially in the initial stage of the disease, being detected in 91.7% of saliva samples from patients with COVID-19 [133–135]. The angiotensin-converting enzyme 2 (ACE2) is known to be the functional SARS-CoV receptor and it plays a crucial role in the pulmonary pathogenesis of SARS [136]. Liu et al. [137] showed, in rhesus monkeys (a study model closer to what occurs in humans), that ACE2+ cells are widely distributed in the upper respiratory tract and that ACE2+ epithelial cells lining the ducts of the salivary glands were the first target cells infected by SARS-CoV-2. This means that the epithelial cells of the salivary glands can be infected *in vivo* shortly after the viral infection, being a source of virus in the saliva, particularly at the beginning of the infection.

Increasing evidence suggests that patients with COVID-19 have several oral health problems, such as dry mouth, blisters on the mucosa, rash in the mouth, lip necrosis, and loss of taste perception and smell recognition [138]. DM, obesity, advanced age, and hypertension are the clinical conditions most associated with SARS-CoV-2 infection or disease progression. It has also been suggested that the periodontal status may indicate the risk for COVID-19 complications [139]. Periodontal disease can also worsen the symptoms associated with COVID-19 and periodontal treatment might help to reduce the symptoms generated by the viral infection. Pro-inflammatory cytokines and oxidative stress, well-known for contributing to the development of periodontal disease and metabolic diseases are highly elevated in SARS-CoV-2 infected individuals. Thus, the relationship between COVID-19 and the oral cavity is still being explored and it has been envisaged that oral manifestations might be used in the early diagnosis and/or onset of the viral infection [138].

Considering that the oral cavity is colonized by a wide variety of microorganisms, viruses are likely to interact with the host microbiota. Patients infected with SARS-CoV-2 may present changes in the oral and intestinal microbiota. Importantly, oral microbial species have been found in the lungs of COVID-19 affected patients [140]. Risk factors such as inadequate oral hygiene, coughing, increased inhalation under normal or abnormal conditions, and mechanical ventilation provide a way for oral microorganisms to reach the lower respiratory tract and, therefore, cause respiratory diseases. For example, pulmonary hypoxia, one of the typical symptoms of COVID-19, favors the growth of anaerobes and facultative anaerobes from the oral microbiota [140]. As the oral microbiome is closely associated with SARS-CoV-2 co-infections, effective oral health care measures are needed to reduce these infections, especially in COVID-19 compromised patients [141]. In addition, multi-omic studies of clinical samples from oral, pulmonary, and intestinal sites are important for better understanding of the role of the host microbiota and its impact on SARS-CoV-2 infection (and vice versa) which will bring new perspectives for the diagnosis, treatment, and prognosis of COVID-19 [140].

The importance of highlighting COVID-19 in this review is due to the fact that DM individuals have a higher mortality rate associated with the infection [142]. Since the initial outbreak of COVID-19, much attention has been focused on DM individuals due to the poor prognosis when these individuals are infected by the virus. The reason for the greater severity of COVID-19 in DM individuals is probably multifactorial, reflecting the syndromic nature of DM. Advanced age, comorbidities such as hypertension and cardiovascular disease, obesity, and a pro-inflammatory and a pro-coagulative state are likely to contribute to the risk of worse outcomes. The virus infection itself can represent a worsening factor for DM individuals, as it can induce acute metabolic complications through direct negative effects on the function of pancreatic β-cells. These effects on the function of these cells can also cause diabetic ketoacidosis and hyperglycemia in individuals with an unknown history of DM or potentially recent DM history [143]. Corona et al. [142] showed that the presence of DM (in individuals aged 60.9 ± 8.2 years) was the best predictor for the mortality rate associated with COVID-19, followed by chronic obstructive pulmonary diseases and the presence of a malignant lesion. Authors also suggest that, due to the induced hyperglycemia in patients infected with Sars-CoV-2, glucose blood levels should be continuously monitored [142].

### Interplay Among DM and Its Risk Factors, the Oral Microbiome, and Oral Cavity Conditions

Among DM-associated risk factors, hyperglycemia, obesity, hypertension, and dyslipidemia will be discussed below. Advanced age and polycystic ovary syndrome are presented in the Supplementary Material.

#### Hyperglycemia

Hemoglobin, which gives the bright red color to blood, is a protein found only in red blood cells. The main function of hemoglobin is to transport oxygen from the lungs to all cells in the body. It becomes glycated or coated with the available glucose in the bloodstream. The hemoglobin A1c test (glycated hemoglobin, glycosylated hemoglobin, HbA1c or A1c) is used to assess the percentage of hemoglobin glycosylation and a person's level of glucose control. In this way, higher A1c levels indicate hyperglycemia and are found in DM patients. For an A1c test to be classified as normal, or in the non-diabetic range, the percentage of glycosylation needs to be below 5.7%. People who present A1c ranging from 5.7 to 6.4% are considered pre-diabetic, and those presenting A1c > 6.5% are considered as diabetic [144]. There is a range of recommendations proposed by the American Diabetes Association (ADA) to improve diabetes outcomes, including strategies to prevent, delay, or effectively manage DM2 and its life-threatening complications. The primary approach involves glycemic control through dietary and lifestyle modifications, blood glucose level monitoring and, depending on the severity of the diabetes, hypoglycemic medications or insulin [145]. It is highly recommended by the ADA that patients with an HbA1C level of 5.7–6.4% receive nutrition advice by a professional dietitian and practice regular physical exercises [145]. A healthy balanced diet includes control of total calories and low/free carbohydrate levels and the consumption of complex dietary fiber and whole grains [145, 146].

Hyperglycemic patients have a longer stay in hospital and a higher rate of stays in the intensive care unit. They are also less likely to be discharged to home and often require transfer to a transitional care unit or nursing home. On hospital admission, hyperglycemia represents an important marker of an unsatisfactory clinical outcome and of mortality in patients with and without a DM history [147]. The assessment of glucose blood levels on hospital admission is of great importance, since infection and hyperglycemia are commonly interconnected. Yi et al. [148] reported the frequency of isolation of nosocomial-related pathogens in patients on admission to the hospital. The authors found that *Enterobacter* spp., *Staphylococcus aureus, Klebsiella pneumoniae, Acinetobacter baumannii, Pseudomonas aeruginosa*, and *Enterococcus faecium* were likely to be found in both normoglycemic and hyperglycemic individuals or DM patients. However, hyperglycemic individuals were more likely to harbor more than one bacterial nosocomial pathogen and the prevalences of multidrug-resistant *K. pneumoniae* and methicillin-resistant *S. aureus* were higher in this group. The authors also found that hyperglycemic individuals presented a higher admission rate to the intensive care unit and a lower survival rate [148].

Glucose is a small molecule that, when found in high levels in the bloodstream can easily diffuse through the semipermeable cell membrane and can therefore be detected in saliva. The presence of glucose in saliva can also be explained due to a process called “diabetic membranopathy” characterized by changes in the basal membrane of the salivary glands and leading to leakage of glucose from the bloodstream into the gland lumen [149]. Carramolino-Cuéllar et al. [150] investigated the correlation between blood and salivary glucose levels in healthy individuals and in DM2 individuals (aged between 40 and 80 years). Samples of venous blood and saliva were collected from both groups on fasting and after the administration of a test meal (containing 15% proteins, 55% carbohydrates, and 30% lipids). Salivary glucose levels were higher in DM individuals compared to the control group, both on fasting and after meals (postprandial period). A significant and positive correlation was also found between blood and salivary glucose levels, especially in DM individuals.

Fares et al. [151] also evaluated the correlation between salivary glucose levels with blood glucose levels and its accuracy in the diagnosis of non-diabetic, diabetic, and pre-diabetic individuals (18-65 years). The mean salivary glucose concentration was 23.40 ± 12.75 mg/dl in non-diabetic, 42.68 ± 20.83 mg/dl in pre-diabetic, and 59.32 ± 19.14 mg/dl in diabetic individuals, being significantly different between all three groups. Although correlations were fair at the best, salivary glucose correlated with blood glucose levels (*r* = 0.67 in non-diabetic individuals, *r* = 0.56 in DM individuals, and *r* = 0.36 in pre-diabetic individuals). Moreover, the salivary glucose level was able to properly differentiate healthy individuals from diabetics and from pre-diabetics, showing a sensitivity of 94.2% and specificity of 62%. The authors concluded that the assessment of salivary glucose levels might be used as a valid and non-invasive test for patient screening as a yield in the diagnosis of DM and pre-diabetic individuals [151]. In addition to this finding, Tiongco et al. [152] showed that amylase levels in unstimulated saliva collected under fasting conditions was higher in DM individuals compared with non-diabetic individuals, suggesting its potential to be used as a salivary marker that helps to diagnose diabetic and non-diabetic individuals. Early diagnosis of DM2 is essential to improve patient prognosis. It has been claimed, based on the above mentioned evidence that the assessment of salivary glucose levels might be a promising alternative to the invasive and painful blood tests for glucose monitoring [153].

Hyperglycemia seems to alter the proportion of specific microorganisms in the oral cavity. Miranda et al. [154] evaluated the influence of glycemic control on the levels of periodontal pathogens (*T.denticola, P. gingivalis, T.forsythia, Eubacterium nodatum, P. micra, F. nucleatum* ssp., and *P. intermedia*) in DM2 patients presenting generalized chronic periodontitis. Subgingival biofilm samples were collected from poor and good glycemic control patients. Levels of *F. nucleatum* ssp. and the detection frequencies of *T. forsythia, E. nodatum, P. micra*, and *F. nucleatum* ssp., were statistically higher in samples from poor glycemic control individuals. Therefore, the authors demonstrated that poor glycemic control is associated with increased levels and frequencies of periodontal pathogens in the subgingival biofilm of individuals with DM2.

Besides these findings, Komazaki et al. [155] analyzed whether the periodontal pathogen *A. actinomycetemcomitans* (Aa), affects glucose metabolism. For this, C57BL/6J mice were administered with Aa or saline solution for 6 weeks and fed with a normal diet or with a high-fat diet. The mice were subjected to glucose and insulin tolerance tests. It was found that the administration of Aa caused impaired glucose tolerance and insulin resistance irrespective of the diet. The authors also showed that insulin resistance was associated with changes in the intestinal microbiota induced by *A. actinomycetemcomitans*. Therefore, these data may suggest a link between periodontal disease, through its periodontal pathogen *A. actinomycetemcomitans*, changes in the intestinal microbiota, and glucose metabolism [155].

#### Obesity

Obesity is a metabolic disease characterized by chronic low-grade inflammation (denominated “meta-inflammation”) which is associated with an imbalance/dysregulation of cellular homeostasis in response to excessive nutrient intake and accumulation [156, 157]. Particularly visceral obesity, which is the accumulation of adipose tissue within the abdominal cavity, is associated with insulin resistance in peripheral glucose and in the use of fatty acids, which can often cause DM2 [158].

Metabolic abnormalities, such as dyslipidemia, hyperinsulinemia, or insulin resistance and obesity, play important roles in the induction and progression of DM2. The study field of immuno-metabolism implies a bidirectional link between the immune system and metabolism, where inflammation plays an essential role in promoting metabolic abnormalities and metabolic factors which, in turn, regulate the functions of immune cells. Obesity, as the main inducer of systemic level inflammation, is one of the main risk factors for DM2. The infiltration of immune cells in adipose tissue of obese individuals, the presence of inflammation, and the increased oxidative stress induce metabolic deficiencies in insulin-sensitive tissues, which are turned into insulin resistant tissues [35].

Adipose tissue is no longer considered an inert tissue functioning only as an energy storing tissue, but is emerging as an important factor in the regulation of many pathological processes [158]. Obesity is associated with a process of chronic inflammation due to the infiltration of multiple immune cells in adipose tissue. These cells include monocytes, macrophages, natural killer cells, and lymphocytes, which result in the secretion of inflammatory cytokines both by the adipocytes present in the tissue and by these populations of infiltrating immune cells [159]. The inflammatory mediators produced by adipose tissue are called adipokines. Among them are adiponectin, leptin, resistin, and visfatin which are an important link between obesity, insulin resistance, and inflammatory diseases [158]. As adipokines play important roles in maintaining energy homeostasis, appetite, glucose and lipid metabolism, insulin sensitivity, angiogenesis, immunity, and inflammation, there is evidence showing that adipokines are involved in the pathogenesis of diseases that affect almost all body systems, including DM, kidney disease, gynecological diseases, rheumatological disorders, cancer, Alzheimer's, depression, muscle disorders, liver disorders, and cardiovascular and lung diseases [160]. Adiponectin and leptin are the most abundant adipokines produced by adipocytes [158]. Individuals with mutations or deficiencies in the leptin gene have low levels of circulating leptin and present extreme obesity. Adiponectin production, on the other hand, can contribute to increased insulin sensitivity [161]. The decrease in adiponectin blood levels is associated with chronic inflammation and metabolic disorders, including DM2 and atherosclerosis [162]. The co-treatment of adiponectin and leptin normalizes the action of insulin in lipoatrophic animals resistant to insulin [161]. However, other immunological mediators can also be produced by adipose tissue which include, among others, TNF-α and IL-6. Both adipocytes and other immunological mediators are responsible for adipose tissue having been redefined as a key component not only of the endocrine system, but also of the immune system [158]. The production of these mediators can be elevated and their expression dysregulated in obese individuals, contributing to insulin resistance. Therefore, crosstalk between adipocytes and the populations of immune cells that infiltrate adipose tissue may govern homeostasis under physiological conditions but contribute to the establishment of chronic subclinical inflammation during obesity development, a prerequisite for insulin resistance [163]. Among the populations of immune cells that infiltrate adipose tissue, macrophages are the main source of adipose tissue-produced TNF-α and these cells contribute to approximately 50% of secreted IL-6. Obese individuals contain a greater number of macrophages compared to the number of these cells found in normal-weight individuals, and these macrophages appear to be activated, from both a morphological (giant cell) and functional (cytokine production) point of view [164].

Macrophages are cells that perform a series of functions, among them, the regulation of inflammatory responses [165]. In meta-inflammation, there is chronic activation of macrophages, which significantly alter the body's inflammatory balance. Since obesity and inflammation were associated, studies show how macrophages assume differential states of activation under the influence of meta-inflammation. Moreover, the role of these cells in maintaining a chronic meta-inflammation environment, which stimulates physiological imbalance, and the development of metabolic syndrome, including DM2, has also been shown [157]. “M0” macrophages are cells originated from hematopoietic progenitors and, depending on the stimulus, these cells can differentiate into other subtypes with different response patterns. “M1” or “classic activated” macrophages originate through a stimulus with LPS, IFN-γ, or TNF-α and these macrophages are characterized by producing pro-inflammatory mediators, such as TNF-α, IL-6, IL-1β, and nitric oxide (NO). The “M2” or “alternatively activated” macrophages are cells derived from stimulation by IL-4, IL-10, IL-13, or glucocorticoids, these macrophages being able to secrete anti-inflammatory mediators, such as IL-4 and IL-10 [166]. Diet-induced obesity leads to a change in the activation state of adipose tissue macrophages from a polarized M2 state in lean animals to a pro-inflammatory M1 state in obese animals, which contributes to insulin resistance. It appears that in diet-induced obesity, there is a decrease in gene expression for M2 and an increase in the gene expression for M1 macrophages [167]. Therefore, adipose tissue macrophages play an active role in obesity and the inflammatory activity related to these cells may contribute to the pathogenesis of obesity-induced insulin resistance [168].

In addition, there are indicators that T cells are important mediators of inflammation in DM2. In obesity and DM2 conditions, an imbalance in Th17 cells and Treg cells is also observed [169]. As a result, there is a loss of T cell homeostasis, contributing to inflammation and tissue and systemic immunity in DM2 [156, 170]. Treg cells are highly present in the adipose tissue of normal-weight mice, while their numbers are extremely low in this same tissue location in insulin-resistant and obese animals. Cytokines secreted by Treg and by fat-resident cells directly affect the synthesis of inflammatory mediators and glucose uptake by adipocytes [171]. An appropriate balance between pro-inflammatory (Th1 and Th17) and anti-inflammatory (Treg and Th2) subsets of T cells is essential to maintain homeostasis and prevent inflammatory diseases [171, 172]. Studies show that inflammation and insulin resistance are related to an increase in the Th17 and Th1 subsets, with a decrease in the Treg subset [171, 172]. DM2 patients have elevated serum levels of IL-6, IL-1β, and TGF-β, the three cytokines known to induce Th17 differentiation [173, 174]. Jagannathan-Bogdan et al. [172] showed that patients with DM2 have a high amount of circulating Th17 cells and an increase in IFN-γ production, showing that T cells are naturally directed to pro-inflammatory subsets, which can be responsible for promoting chronic inflammation in DM2 due to the high production of pro-inflammatory cytokines. Therefore, Th17, depending on the microenvironment, can form pathogenic and non-pathogenic subpopulations and, in obesity, the pool of inflammatory pathogenic Th17 cells with cytotoxic potential is also responsible for the development of accompanying autoimmune reactions [156].

As detailed earlier in this article, DM1 is associated with the loss of β-pancreatic cells from the islets of Langerhans resulting in the inability of the cells to produce the insulin needed to maintain body glycemic control. It has also been reported that Th17 cells have contributed to DM disease progression due to this cellular profile, stimulating the production of pro-inflammatory cytokines, recruiting and activating neutrophils and macrophages, and also neutralizing Treg functions that regulate the immune response. Thus, it is suggested that the main function of IL-17 is to increase inflammatory responses and autoimmune destruction of β-pancreatic cells responsible for insulin production in the pancreas [175]. In addition to destroying these cells, IL-17 can also cause complications in other organs such as the kidneys and intestine [176]. Therefore, blocking differentiation of Th17 cells is now considered one of the potential therapeutic strategies for DM1 and DM2 management, although the application of this immunotherapy needs to be better evaluated, mainly in cases of DM1, which presents an extremely complex immunological condition [175, 176].

Overweight and obesity, besides representing a strong risk factor for other health complications, can also interfere with the oral microbiota. Goodson et al. [177] investigated the salivary microbial composition of overweight women (body mass index between 27 and 32). The authors found that approximately 98.4% of overweight women could be identified by the presence of a single bacterial species *S. noxia*, representative of the phylum Firmicutes. Moreover, saliva of overweight individuals presented a greater median percentage of Firmicutes (*V. parvula, S. mitis, S. anginosus, S. oralis, S. gordonii, S. intermedius, S. sanguinis, Peptostreptococcus micros, Streptococcus constellatus*, Bacteroidetes (*P. melaninogenica, P. intermedia, P. nigrescens, Capnocytophaga ochracea, P. gingivalis, Capnocytophaga sputigena, T. forsythia, C. gingivalis*), Proteobacteria (*Neisseria mucosa A. actinomycetemcomitans, C. rectus*). Fusobacteria (*Fusobacterium periodonticum, F.nucleatum*), Actinobacteria (*A. viscosus, A. gerencseriae, A. naeslundii, Actinomyces israelii, P. acnes*), and Spirochaetae (*Treponema socranskii, T. denticola*). Moreover, Tam et al. [178] investigated whether obesity plays a role in modulating oral microbial composition and diversity. In that study, subgingival dental biofilm and saliva were collected from 18 patients with DM2, including 6 obese (body mass index ≥ 30 kg/m^2^) and 12 normal-weight patients. Most of the participants presented moderate or severe forms of periodontitis. A greater abundance of Bacteroidetes, Spirochaetes, Firmicutes, *Treponema* spp., *Selenomomas* spp., and *Filifactor* spp. was found in the subgingival biofilm of normal-weight individuals, while the abundance of Proteobacteria, Firmicutes, *Chloroflexi* spp., and *Campylobacter* spp. was greater and Bacteroidetes were virtually absent in obese individuals. Similar results were found in saliva: a greater abundance of Bacteroidetes and Firmicutes in normal-weight individuals, with Firmicutes being more abundant in obese individuals. The differences in microbial composition and diversity between the obese and normal-weight individuals were statistically significant, indicating reduced species diversity in the obese individuals [178].

Janem et al. [179] showed that DM2 adolescents (aged 10–19 years) tend to present worse gingival conditions (in terms of gingival inflammation) than obese and normal-weight individuals. However, *Fretibacterium* spp. was exclusively found in saliva of DM2 individuals who also showed a lower abundance of *Haemophilus* spp., *Alloprevotella* spp., *Pseudomonas* spp., and *Lautropia* spp. compared with the other groups. *Cardiobacterium* spp. and *Corynebacterium* spp. were more abundant in saliva of obese individuals, while *Pseudomonas* spp. was more abundant in saliva of normal-weight individuals. Moreover, obese DM2 adolescents tend to present worse oral health conditions compared with normal-weight adolescents and with obese adolescents who do not present DM2. In order to assess the relationship between salivary microbiota composition, gingival health status, and overweight in adolescents, Araujo et al. [180] analyzed 248 adolescents (14–17 years), without dental caries and without periodontitis. The authors found higher percentages of *S. mutans* and *Bifidobacterium* spp. in overweight and obese individuals compared with normal-weight individuals. No differences were found in the frequency of gingivitis, or percentages of *P. gingivalis* and *Streptococcus pneumoniae* among the groups. In addition, a positive relationship was observed between the accumulation of body fat and salivary counts of *Bifidobacterium* spp., indicating a possible interaction between oral microbial communities and weight gain [180].

#### Hypertension

Hypertension is among the most common chronic medical conditions, characterized by a persistent rise in blood pressure. The current clinical parameter for diagnosing hypertension is systolic blood pressure values of 130 mmHg or more and/or diastolic blood pressure above 80 mmHg [181]. In order to reduce the risk of stroke, it has been recommended that the blood pressure of DM patients be maintained between 120 and 130/80 mmHg [182].

Inflammation, bacteremia, immune response, and metabolic syndrome are specific conditions considered as classic risk factors associated with the development of periodontal disease. Periodontal pathogens can invade the blood vessel wall and trigger an inflammatory response inducing an endothelial dysfunction. In hypertension, changes in microcirculation can cause ischemia in the periodontium, favoring periodontal disease. This seems to act as a common link to explain the relationship between hypertension and periodontal disease [183]. In this context, Vidal et al. [184] showed that periodontal therapy significantly reduced blood pressure in refractory hypertension individuals who presented periodontitis (aged 53.6 ± 8.0 years), decreasing cardiovascular risk in hypertensive patients. Pietropaoli et al. [185] assessed the relationship between systemic exposure to periodontal microbiota (indirectly measured by specific blood antibodies) and blood pressure. The presence of antibodies against *C. rectus, V. parvula*, and *P. melaninogenica* was consistently associated with elevated or uncontrolled blood pressure. Moreover, the presence of gingival bleeding seems to increase the chance of adults aged 30 years presenting uncontrolled or high blood pressure by 42% [186]. The study of Czesnikiewicz-Guzik et al. [187] showed that hypertension can also be mediated by immunological mechanisms. The authors used *P.gingivalis* antigens to induce a Th1 immune response in C57BL/6J mice and found that exposure to *P. gingivalis* antigens increased immune activation of T cells that led to aortic vascular inflammation. These immunological changes were associated with increased blood pressure and endothelial dysfunction, revealing another important link between periodontitis and hypertension [187].

Hypertension is also associated with decreased production and bioavailability of NO in the bloodstream. In this sense, the reestablishment of NO levels by nitrite and nitrate has been considered a potential therapeutic strategy for the treatment of this condition [188]. NO participates in a large number of physiological functions, including vasodilation, nerve transmission, host defense, metabolism, and mitochondrial function. NO is generated endogenously by a family of specific enzymes called NO synthases (NOS) and also by an alternative pathway which is the nitrate-nitrite-NO pathway. In the first pathway, NOS catalyze the oxidation of the amino acid L-arginine in the presence of O_2_ and various cofactors. Nitrate and nitrite are oxidation products of NOS-dependent NO generation, but also constituents of our diet [189]. Nitrate is obtained by eating green leafy vegetables such as lettuce or spinach [190], while nitrite can also be ingested through the consumption of cured meats and bacon, since nitrite is used as a preservative in these foods. However, in the mouth, the nitrate obtained from the diet can also be reduced to nitrite by oral commensal bacteria [189, 191]. Some oral bacteria found on the posterior part of the dorsum of the tongue, such as *Neisseria* spp., *Prevotella* spp., *Rothia* spp., and *Veillonella* spp. can efficiently reduce nitrate into nitrite. Bacteria use nitrate for their respiration process and, at the same time, help the host by converting nitrate into NO (in a nitrate-nitrite-NO pathway), since mammalian cells cannot effectively metabolize nitrate. Salivary nitrite is then swallowed and reaches the stomach and intestinal tract where it is efficiently absorbed. Once the nitrite reaches the systemic circulation, there are several enzymatic and non-enzymatic pathways for its reduction into NO and other reactive nitrogen intermediates [189, 191]. In this way, dietary nitrate intake and the metabolism of nitrate-reducing bacteria act synergistically in lowering blood pressure, maintaining and improving cardiovascular health throughout life [192]. Cigarette smoke, mouthwash, and proton pump inhibitors are among the agents known to disrupt the nitrate enterosalivary cycle [188].

In this sense, Hyde et al. [193] observed that 7 days after dietary supplementation with sodium nitrate, the diastolic blood pressure of rats decreased significantly and the abundance of the *Haemophilus* spp. (nitrate reducers) and *Streptococcus* spp. (nitrite reducers) present on tongue dorsum increased. Moreover, Burleigh et al. [194] showed that nitrate dietary supplementation in young individuals (age 27 ± 7 years) decreases blood pressure and increases both salivary and blood nitrite levels. These authors also found that the rate of reduction of nitrate to nitrite was dependent on the abundance of nitrate-reducing bacteria in the oral cavity. Individuals presenting a lower abundance of these bacteria presented a lower nitrite salivary level [194]. Interestingly, Vanhatalo et al. [192] showed that the dietary nitrate supplementation (through consumption of nitrate rich concentrated beetroot juice) over 10 days of administration in young (18–22 years old) and older (70–79 years old) individuals led to an increase in the relative abundance of Proteobacteria (+225%) and a decrease in the abundance of Bacteroidetes (-46%) in saliva. Moreover, nitrate supplementation led to higher nitrite blood levels and a reduction in the blood pressure of the older individuals. High abundances of *Rothia* spp. (+127%) and *Neisseria* spp. (+131%) and low abundances of *Prevotella* spp. (−60%) and *Veillonella* spp. (−65%) were correlated with greater increases in plasma nitrite in response to nitrate supplementation [192]. Velmurugan et al. [195] examined the effects of 6-weeks of daily nitrate intake (nitrate-rich beet juice) on vascular and platelet function of untreated hypercholesterolemia individuals. Dietary nitrate resulted in an increase in vascular function and a reduction in platelet-monocyte aggregates compared to a control group who were administered low-nitrate supplementation. Salivary microbiome composition also changed after nitrate supplementation: 78 bacterial taxa were differently abundant, with an increase in the proportion of *Rothia mucilaginosa* and *Neisseria flavescens* [195].

Considering the role played by oral microbiota (the nitrate reducing ones) on blood pressure control, it has been envisaged that the oral microbiome composition might need to be taken into account during the treatment of arterial hypertension, representing new venues in cardiovascular medicine [196]. All these data show that the composition of the oral microbiome may be changed by dietary nitrate supplementation, with this strategy being associated with vascular health. However, the long-term effects of dietary nitrate supplementation on the clinical outcomes of cardiovascular diseases still need to be investigated [197].

As already mentioned, NO is associated with hypertension and, possibly, with the pathogenesis of cardiovascular diseases, which in turn are associated with obesity and DM. DM2 is the most frequent complication of obesity and, interestingly, the decrease in the bioavailability of NO is a characteristic commonly found in obese and DM individuals. Nitrate and nitrite, in addition to promoting the production of NO, reduce oxidative stress and increase insulin secretion, with these effects being desirable for the control of obesity and DM. Based on current data, it has been suggested that the production of NO from nitrate/nitrite can potentially be used as a nutrition-based therapy in obesity and in DM. The amplification of the nitrate-nitrite-nitric oxide pathway is a desirable strategy to increase the bioavailability of NO [198].

#### Dyslipidemia

Dyslipidemia is characterized by a high concentration of plasma triglycerides, a low concentration of HDL cholesterol, and an increased concentration of small dense particles of LDL cholesterol [199, 200]. These lipid changes are associated with DM. Cholesterol deposits and pro-inflammatory cytokines are involved in the pathogenesis of atherosclerosis, one of the predominant causes of cardiovascular disease. Thus, dyslipidemia and cardiovascular disease are complications commonly found in DM patients. The mortality from coronary heart disease increases exponentially as a result of increased blood cholesterol levels and the reduction in cholesterol levels, with statins seeming to reduce the risk for cardiovascular complications in DM patients [199].

It seems that periodontal pathogens accelerate the growth of atherosclerotic plaque. In the study of Rivera et al. [201], mice deficient in apolipoprotein E and orally infected with *P. gingivalis, T. denticola*, and *T. forsythia* developed aortic plaque with accumulation of macrophages as well as presenting increased amyloid, cholesterol, and triglyceride serum levels. Dissemination of the infection was observed by the identification of bacterial genomic DNA in the aorta and in liver as well as by high levels of bacteria-specific IgG antibodies [201]. In a similar study, Chukkapalli et al. [202] infected the oral cavity of mice (also defective in apolipoprotein E) with *P. gingivalis, T. denticola, T. forsythia*, and *F. nucleatum*. The authors observed dissemination of those microorganisms to the heart and aorta. The presence of this polymicrobial infection also induced higher levels of oxidized LDL and accelerated aortic plaque formation.

Choi et al. [203] investigated, through a cross-sectional study, the relationship between the prevalence and the total load of periodontal bacteria in the oral cavity and the serum lipid profile. Saliva samples from older adults (age of 69.2 years) were collected and assessed to identify *P. gingivalis, T. denticola, T. forsythia*, and *P. intermedia*. Triglycerides and HDL cholesterol were quantified in blood samples. The number of bacterial species and their coexistence with periodontitis were significantly related to a decrease in HDL and an increase in triglyceride blood levels. The adjusted mean levels of HDL in individuals with low, moderate, and high levels of bacterial species were 66.1, 63.0, and 58.9 mg/dL, respectively. The data obtained provide evidence that the load of periodontal pathogens in saliva might interfere with the regulation of HDL and triglyceride blood levels, which are critical factors for atherosclerosis/cardiovascular disease. Moreover, viable periodontal pathogens, including *P. gingivalis*, have been found in atherosclerotic plaques [204–206]. Køllgaard et al. [206] showed that incubation of human monocytes with cholesterol crystals induced the secretion of inflammatory mediators IL-1β, TNF-α, IL-6, and IL-8 (pro-atherogenic cytokines). In addition, cholesterol crystals also increased the secretion of IL-1β in the presence of both *P. gingivalis* and its derived LPS. These data suggest that cholesterol and *P. gingivalis* synergize to promote the production of pro-atherogenic cytokines.

In addition to the involvement of periodontal pathogens, *S. mutans*, a supragingival biofilm colonizer, has also been detected in atherosclerotic plaque. Kesavalu et al. [207] showed that mice deficient in apolipoprotein E and orally infected with *S. mutans* developed atherosclerotic plaque. Furthermore, systemic dissemination of *S. mutans* was observed, since genomic DNA from these bacteria was detected in the aorta, liver, spleen, and heart. An increased number of macrophages was also found in the aorta. These data suggest that *S. mutans* might also be associated with the growth of atherosclerotic plaques [207].

## Concluding Remarks

We show in this review that DM, in individuals with poorly controlled glycemic index, may have a negative effect on the immune system of affected individuals, leading to a deficient immune response or, on the other hand, there may be an exacerbation of the inflammatory response in these individuals which could potentially cause an exacerbation of DM-related complications. Among these complications, individuals affected by DM seem to be more susceptible to infectious diseases, presenting more severe infectious courses and greater morbidity and mortality compared to non-diabetic ones.

**Figure 1:**
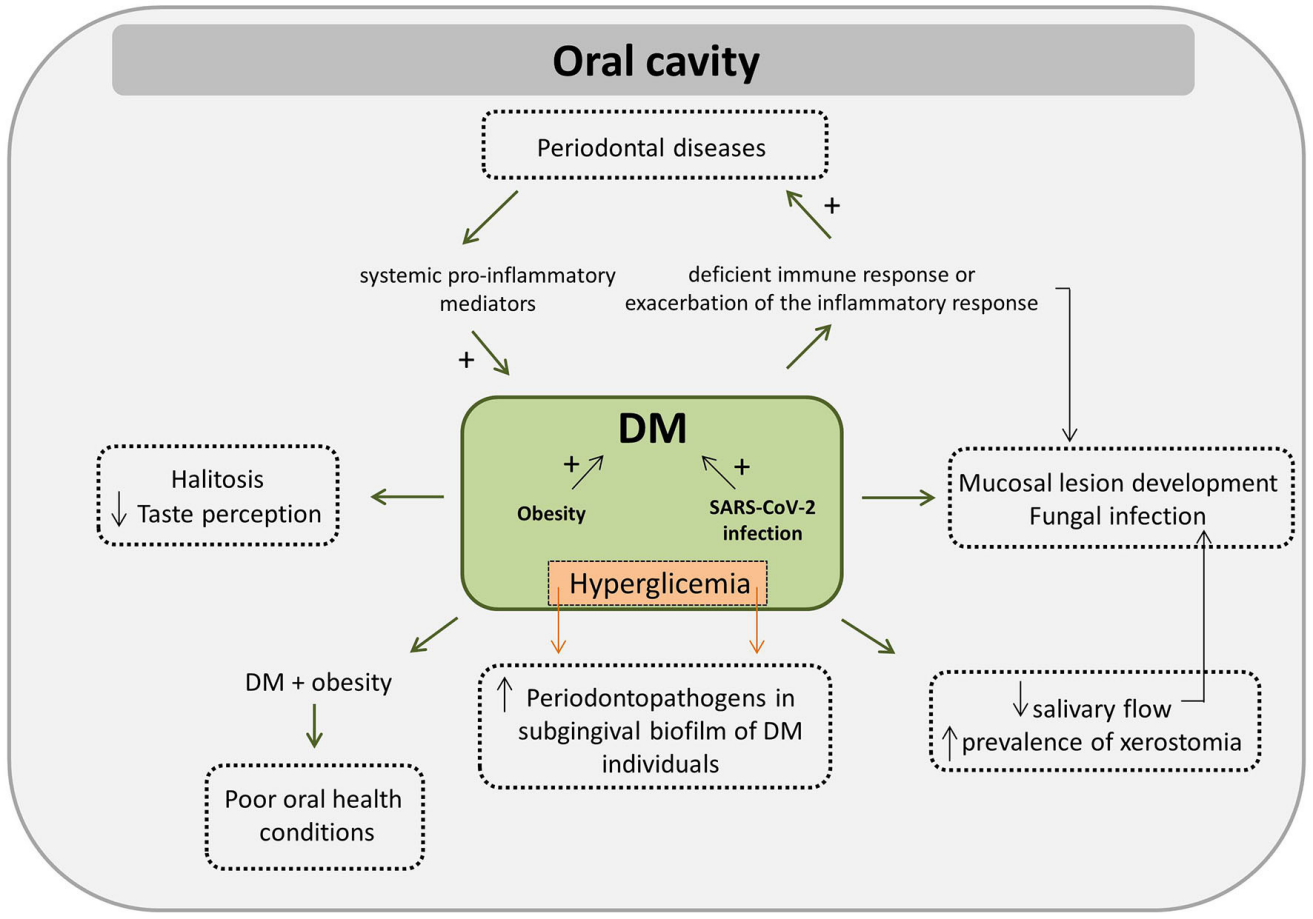
Interplay among oral cavity conditions, host immune response, DM and some of its risk factors. DM, Diabetes mellitus, “+” means aggravating condition.

Regarding oral conditions, individuals with DM tend to have low salivary flow and a high prevalence of xerostomia (Figure 1). A significant and positive correlation between blood and salivary glucose levels has also been suggested, especially in individuals with DM where high levels of salivary glucose can create an acidic environment in the oral cavity which, in turn, promotes changes in the salivary microbiota compared with non-diabetic individuals. Some microorganisms, such as fungi, adhere more to epithelial and endothelial cells also in glucose-rich environments compatible with hyperglycemia. The association between prevalence/experience of dental caries and DM is still unclear. While some studies indicate a higher prevalence of caries in individuals with uncontrolled blood glucose levels, other studies did not find an association between dental caries and DM due to different levels of glycemic control and heterogeneity in age and in the level of HbA1c among the individuals studied.

A bidirectional relationship between periodontal diseases and DM has been suggested as a consequence of the inflammatory characteristic of both diseases. On one hand, DM patients may have an exaggerated inflammatory response, poor repair and bone resorption that aggravates periodontal disease, leading to tooth loss. On the other hand, the increased levels of systemic pro-inflammatory mediators found in individuals affected with periodontal disease exacerbates insulin resistance (Figure 1). Studies also show that there is a reduction in salivary microbial diversity and in the richness and diversity of the subgingival biofilm microbiota in individuals with DM, which seem to be more susceptible to alterations in the subgingival microbiome toward dysbiosis. Evidence suggests that periodontal therapy might significantly contribute to glycemic control in DM patients.

Some mucosal manifestations seem to be frequent in DM individuals. DM has also been associated with the development of lesions of the oral mucosa. There is a higher prevalence of changes in the normal appearance of the oral mucosa, especially in relation to potentially malignant diseases and lesions associated with fungal infections, in individuals with DM (Figure 1). Patients with DM and oral cancer have higher mortality when compared to non-diabetics with oral cancer.

Pro-inflammatory cytokines and oxidative stress, well known to contribute to the development of periodontal and metabolic diseases, are highly elevated in individuals infected with SARS-CoV-2, which could indicate an important relationship between these conditions. DM (and some of its associated risk factors, such as obesity, advanced age and hypertension) is the clinical conditions most associated with infection or disease progression by SARS-CoV-2 (Figure 1). The virus infection itself may represent an aggravating factor for individuals with DM, as it can induce acute metabolic complications through direct negative effects on the function of pancreatic β cells. An association between oral cavity, COVID-19 and DM has not yet been demonstrated. Since periodontal disease is associated with DM, and considering the association between each of these conditions with COVID-19, it has been speculated that individuals co-affected by periodontal diseases and DM may present more COVID-19-related aggravated complications. This, in turn, needs to be confirmed, or not, by clinical studies.

Hyperglycemia induces alterations in the oral microbiome since poor glycemic control is associated with increased levels and frequencies of periodontal pathogens in the subgingival biofilm of individuals with DM (Figure 1). It has been shown that periodontopathogens may induce changes in the gut microbiota, which, in turn, may lead to the development of insulin resistance. In terms of general health, infection and hyperglycemia are commonly intertwined conditions. Hyperglycemic patients have a longer hospital stay and a higher rate of stay in the intensive care unit.

Visceral obesity is associated with insulin resistance. Obesity plays an important role in the induction and progression of DM since it also promotes a chronic low-grade inflammation (meta-inflammation) (Figure 1). In obesity and DM conditions, an imbalance of Th17 cells and Treg cells is also observed. Loss of T cell homeostasis contributes to inflammation and to tissue and systemic immunity in DM. Abundance of specific bacterial genera on saliva and on subgingival biofilm of obese individuals is different compared with normal-weight individuals, indicating a reduced microbial diversity on oral cavity of obese individuals. A positive relationship was observed between the accumulation of body fat and salivary counts of *Bifidobacterium* spp., indicating a possible interaction between oral microbial communities and weight gain. Co-affected obese and DM individuals tend to present worse oral health conditions (Figure 1). Evidences also suggest that obesity induces differential states of activation on macrophages cells which turn into a pro-inflammatory. At this state, macrophages play an active role in obesity and the inflammatory activity related to these cells may contribute to the pathogenesis of obesity-induced insulin resistance.

Several microorganisms from the posterior part of the dorsum of the tongue are able to reduce nitrate into nitrite. Dietary nitrate intake and the metabolism of nitrate-reducing bacteria act synergistically in lowering blood pressure, maintaining and improving cardiovascular health throughout life. The composition of the oral microbiome may change by dietary nitrate supplementation being this strategy associated with vascular health. An important interplay among oral cavity microbiome, DM, obesity, and hypertension has been proposed as the reduction of nitrate into nitrite, in addition to contribute to lowering of blood pressure, reduces oxidative stress and increases insulin secretion, being these effects desirable for the control of obesity and DM.

Dyslipidemia is also associated with DM. Cholesterol deposits and pro-inflammatory cytokines are involved in the pathogenesis of atherosclerosis. This way, dyslipidemia and cardiovascular disease are complications commonly found in DM patients. A decrease in HDL and, an increase in triglycerides bloodstream levels seem to be associated with an increase on the load of periodontopathogens on oral cavity. Conversely, the load of periodontal pathogens in saliva might interfere with the regulation of HDL and triglycerides blood levels. Cholesterol and periodontal pathogens (especially *P. gingivalis*) synergize to promote the production of pro-atherogenic cytokines accelerating the growth of atherosclerotic plaque.

Based on the data available in the Supplementary Material, we may highlight that:

Production of volatile sulfur compounds by microorganisms found in the dorsum of the tongue and/or in the periodontal pockets of patients presenting periodontal disease is considered the most frequent factors associated with intra-oral origin halitosis. Some studies even consider the presence of DM as a factor that increases the likelihood of halitosis. Additionally, breathe concentration of volatile organic compounds, especially ketone bodies, are also associated with halitosis. Insufficient insulin secretion is associated with increased ketone bodies on exhaled air in DM affected individuals.

Prevalence of impaired taste perception (especially to salt) and impaired smell recognition tend to be greater in DM patients and this sensory impairment seems to be associated with higher fasting glycemia in the affected individuals. The mechanism involved in this sensory impairment found in DM individuals is still not completely understood.

Among the DM associated risk factors, evidences suggest that advanced age might be associated with a higher prevalence of edentulism especially in individuals with multimorbidity including DM. Microbial changes associated with aging seems to be associated with the increase in the prevalence and severity of periodontitis, but a direct relationship among DM, oral cavity microbiome and advanced age was not found.

DM and PCOS are commonly found as associated conditions. PCOS is also often associated with abdominal adiposity, insulin resistance, metabolic disorders and cardiovascular risk factors. Women with PCOS tend to present a distinct oral microbial composition and an elevated systemic response to selective members of this microbial community, but the association between oral microbiome, PCOS are DM is still unknown.

## Author Contributions

RA and TN contributed to conception and design. All authors drafted the manuscript, contributed to manuscript revision, read, and approved the submitted version.

## Conflict of Interest

The authors declare that the research was conducted in the absence of any commercial or financial relationships that could be construed as a potential conflict of interest.

## Publisher's Note

All claims expressed in this article are solely those of the authors and do not necessarily represent those of their affiliated organizations, or those of the publisher, the editors and the reviewers. Any product that may be evaluated in this article, or claim that may be made by its manufacturer, is not guaranteed or endorsed by the publisher.

## References

[1] Dewhirst FE Chen T Izard J Paster BJ Tanner ACR Yu. et al. The human oral microbiome. J Bacteriol. (2010) 192:5002–17. 10.1128/JB.00542-10 20656903 PMC2944498

[2] Lederberg J McCray AT. ‘Ome Sweet' Omics-a genealogical treasury of words. Scientist. (2001) 15:8.

[3] Øilo M Bakken V. Biofilm and dental biomaterials. Materials. (2015) 8:2887–900. 10.3390/ma8062887

[4] Kilian M Chapple I Hannig M Marsh PD Meuric V Pedersen AML. The oral microbiome - an update for oral healthcare professionals. Brit Dent. J. (2016) 221:657–66. 10.1038/sj.bdj.2016.865 27857087

[5] Takahashi N. Oral microbiome metabolism: from “Who are they?” to “What are they doing? J Dent Res. (2015) 94:1628–37. 10.1177/0022034515606045 26377570

[6] Escapa IF Chen T Huang Y Gajare P Dewhirst FE Lemon KP. New insights into human nostril microbiome from the expanded human oral microbiome database (eHOMD): a resource for the microbiome of the human aerodigestive tract. mSystems. (2018) 3:e00187–18. 10.1128/mSystems.00187-18 30534599 PMC6280432

[7] Rasiah IA Wong L Anderson SA Sissons CH. Variation in bacterial DGGE patterns from human saliva: over time, between individuals and in corresponding dental plaque microcosms. Arch Oral Biol. (2005) 50:779–87. 10.1016/j.archoralbio.2005.02.001 15970209

[8] Belstrom D Holmstrup P Bardow A Kokaras A Fiehn NE Paster BJ. Temporal stability of the salivary microbiota in oral health. PLoS ONE. (2016) 11:e0147472. 10.1371/journal.pone.0147472 26799067 PMC4723053

[9] Baquero F Nombela C. The microbiome as a human organ. Clin Microbiol Infect. (2012) 4:2–4. 10.1111/j.1469-0691.2012.03916.x 22647038

[10] Roberts FA Darveau RP. Microbial protection and virulence in periodontal tissue as a function of polymicrobial communities: symbiosis and dysbiosis. Periodontol 2000. (2015) 69:18–27. 10.1111/prd.12087 26252399 PMC4530467

[11] De Filippis F Vannini L La Storia A Laghi L Piombino P Stellato G. The same microbiota and a potentially discriminant metabolome in the saliva of omnivore, ovo-lacto-vegetarian and vegan individuals. PLoS ONE. (2014) 9:e112373. 10.1371/journal.pone.0112373 25372853 PMC4221475

[12] Takeshita T Kageyama S Furuta M Tsuboi H Takeuchi K Shibata Y. Bacterial diversity in saliva and oral health-related conditions: the Hisayama study. Sci Rep. (2016) 6:22164. 10.1038/srep22164 26907866 PMC4764907

[13] Zaura E Brandt BW Prodan A Teixeira de Mattos MJ Imangaliyev S Kool J. On the ecosystemic network of saliva in healthy young adults. ISME J. (2017) 11:1218–231. 10.1038/ismej.2016.199 28072421 PMC5475835

[14] Willis JR González-Torres P Pittis AA Bejarano LA Cozzuto L Andreu-Somavilla N. Citizen science charts two major “stomatotypes” in the oral microbiome of adolescents and reveals links with habits and drinking water composition. Microbiome. (2018) 6:218. 10.1186/s40168-018-0592-3 30522523 PMC6284318

[15] Willis JR Gabaldón T. The human oral microbiome in health and disease: from sequences to ecosystems. Microorganisms. (2020) 8:308. 10.3390/microorganisms8020308 32102216 PMC7074908

[16] Peters BA Wu J Hayes RB Ahn J. The oral fungal mycobiome: characteristics and relation to periodontitis in a pilot study. BMC Microbiol. (2017) 17:157. 10.1186/s12866-017-1064-9 28701186 PMC5508751

[17] Pérez-Brocal V Moya A. The analysis of the oral DNA virome reveals which viruses are widespread and rare among healthy young adults in Valencia (Spain). PLoS ONE. (2018) 13:e0191867. 10.1371/journal.pone.0191867 29420668 PMC5805259

[18] Wade WG. The oral microbiome in health and disease. Pharmacol Res. (2013) 69:137–43. 10.1016/j.phrs.2012.11.006 23201354

[19] Verma D Garg PK Dubey AK. Insights into the human oral microbiome. Arch Microbiol. (2018) 200:525–40. 10.1007/s00203-018-1505-3 29572583

[20] Zhang Y Wang X Li H Ni C Du Z Yan F. Human oral microbiota and its modulation for oral health. Biomed Pharmacother. (2018) 99:883–93. 10.1016/j.biopha.2018.01.146 29710488

[21] Suárez LJ Garzón H Arboleda S Rodríguez A. Oral dysbiosis and autoimmunity: from local periodontal responses to an imbalanced systemic immunity. Front Immunol. (2020) 11:591255. 10.3389/fimmu.2020.591255 33363538 PMC7754713

[22] Pessin JE Saltiel AR. Signaling pathways in insulin action: molecular targets of insulin resistance. J Clin Invest. (2000) 106:165–169. 10.1172/JCI10582 10903329 PMC314316

[23] Tyagi A Pugazhenthi S. Targeting insulin resistance to treat cognitive dysfunction. Mol Neurobiol. (2021) 58:2672–91. 10.1007/s12035-021-02283-3 33483903 PMC9196144

[24] Kullmann S Heni M Hallschmid M Fritsche A Preissl H Häring HU. Brain insulin resistance at the crossroads of metabolic and cognitive disorders in humans. Physiol Rev. (2016) 96:1169–209. 10.1152/physrev.00032.2015 27489306

[25] Milstein JL Ferris HA. The brain as an insulin-sensitive metabolic organ. Mol Metab. (2021) 101234. 10.1016/j.molmet.2021.101234 33845179 PMC8513144

[26] Scherer T Sakamoto K Buettner C. Brain insulin signalling in metabolic homeostasis and disease. Nat Rev Endocrinol. (2021) 17:468–83. 10.1038/s41574-021-00498-x 34108679

[27] Kellar D Craft S. Brain insulin resistance in Alzheimer's disease and related disorders: mechanisms and therapeutic approaches. Lancet Neurol. (2020) 19:758–66. 10.1016/S1474-4422(20)30231-3 32730766 PMC9661919

[28] American Diabetes Association. Classification and Diagnosis of Diabetes: standards of medical care in Diabetes - 2020. Diabetes. Care. (2020) 1:S14–31. 10.2337/dc20-S002 31862745

[29] DiMeglio LA Evans-Molina C Oram RA. Type 1 diabetes. Lancet. (2018) 391:2449–62. 10.1016/S0140-6736(18)31320-5 29916386 PMC6661119

[30] Tang W Liang H Cheng Y Yuan J Huang G Zhou Z. Diagnostic value of combined islet antigen-reactive T cells and autoantibodies assays for type 1 diabetes mellitus. J Diabetes Investig. (2021) 12:963–9. 10.1111/jdi.13440 33064907 PMC8169367

[31] American Diabetes Association. Diagnosis and classification of diabetes mellitus. Diabetes Care. (2013) 1:S67–74. 10.2337/dc13-S067 23264425 PMC3537273

[32] Rachdaqui N. Insulin: the friend and the foe in the development of type 2 diabetes mellitus. Int J Mol Sci. (2020) 21:1770. 10.3390/ijms21051770 32150819 PMC7084909

[33] MacDonald MJ Longacre MJ Langberg EC Tibell A Kendrick MA Fukao T. Decreased levels of metabolic enzymes in pancreatic islets of patients with type 2 diabetes. Diabetologia. (2009) 52:1087–91. 10.1007/s00125-009-1319-6 19296078 PMC2903059

[34] Fletcher B Gulanick M Lamendola C. Risk factors for type 2 diabetes mellitus. J Cardiovasc Nurs. (2002) 16:17–23. 10.1097/00005082-200201000-00003 11800065

[35] Daryabor G Atashzar MR Kabelitz D Meri S Kalantar K. The effects of type 2 diabetes mellitus on organ metabolism and the immune system. Front Immunol. (2020) 11:1582. 10.3389/fimmu.2020.01582 32793223 PMC7387426

[36] Saravanan P. Diabetes in pregnancy working group gestational diabetes: opportunities for improving maternal and child health. Lancet Diabetes Endocrinol. (2020) 8:793–800. 10.1016/S2213-8587(20)30161-3 32822601

[37] American Diabetes Association. Diagnosis and classification of diabetes mellitus. Diabetes Care. (2014) 1:S81–90. 10.2337/dc14-S081 24357215

[38] World Health Organization. Diabetes. (2020). Available online at: https://www.who.int/health-topics/diabetes#tab=tab_1 (accessed April 14, 2021).

[39] Toniolo A Cassani G Puggioni A Rossi A Colombo A Onodera T. The diabetes pandemic and associated infections: suggestions for clinical microbiology. Rev Med Microbiol. (2019) 30:1–17. 10.1097/MRM.0000000000000155 30662163 PMC6319590

[40] Pivari F Mingione A Brasacchio C Soldati L. Curcumin and type 2 diabetes mellitus: prevention and treatment. Nutrients. (2019) 11:1837. 10.3390/nu11081837 31398884 PMC6723242

[41] Poudel P Griffiths R Wong VW Arora A Flack JR Khoo CL. Oral health knowledge, attitudes and care practices of people with diabetes: a systematic review. BMC Public Health. (2018) 18:577. 10.1186/s12889-018-5485-7 29716561 PMC5930945

[42] Verhulst MJL Loos BG Gerdes VEA Teeuw WJ. Evaluating all potential oral complications of diabetes mellitus. Front Endocrinol. (2019) 10:56. 10.3389/fendo.2019.00056 30962800 PMC6439528

[43] Graves DT Kayal RA. Diabetic complications and dysregulated innate immunity. Front Biosci. (2008) 13:1227–39. 10.2741/2757 17981625 PMC3130196

[44] Ochoa-González FL González-Curiel IE Cervantes-Villagrana AR Fernández-Ruiz JC Castañeda-Delgado JE. Innate immunity alterations *in type 2 diabetes* mellitus: understanding infection susceptibility. Curr Mol Med. (2020) 21:318–31. 10.2174/1566524020999200831124534 32867637

[45] Jafar N Edriss H Nugent K. The effect of short-term hyperglycemia on the innate immune system. Am J Med Sci. (2016) 351:201–11. 10.1016/j.amjms.2015.11.011 26897277

[46] Kuwabara WMT Yokota CNF Curi R Alba-Loureiro TC. Obesity and type 2 diabetes mellitus induce lipopolysaccharide tolerance in rat neutrophils. Sci Rep. (2018) 8:17534. 10.1038/s41598-018-35809-2 30510205 PMC6277411

[47] Stegenga ME van der Crabben SN Blümer RME Levi M Meijers JCM Serlie MJ. Hyperglycemia enhances coagulation and reduces neutrophil degranulation, whereas hyperinsulinemia inhibits fibrinolysis during human endotoxemia. Blood. (2008) 112:82–9. 10.1182/blood-2007-11-121723 18316629 PMC2435690

[48] Lowry SF. Human endotoxemia: a model for mechanistic insight and therapeutic targeting. Shock. (2005) 24:94–100. 10.1097/01.shk.0000191340.23907.a1 16374380

[49] Geerlings SE Hoepelman AI. Immune dysfunction in patients with diabetes mellitus (DM). FEMS Immunol Med Microbiol. (1999) 26:259–65. 10.1111/j.1574-695X.1999.tb01397.x 10575137

[50] Critchley JA Carey IM Harris T DeWilde S Hosking FJ Cook DG. Glycemic control and risk of infections among people with type 1 or type 2 diabetes in a large primary care cohort study. Diabetes Care. (2018) 41:2127–35. 10.2337/dc18-0287 30104296

[51] Cockram CS Wong BCK. Diabetes and infections. Diabetes. (2017) 55, 799–818. 10.1002/9781118924853.ch55

[52] Alba-Loureiro TC Munhoz CD Martins JO Cerchiaro GA Scavone C Curi R. Neutrophil function and metabolism in individuals with diabetes mellitus. Braz J Med Biol Res. (2007) 40:1037–44. 10.1590/S0100-879X2006005000143 17665039

[53] Indyk D Bronowicka-Szydełko A Gamian A Kuzan A. Advanced glycation end products and their receptors in serum of patients with type 2 diabetes. Sci Rep. (2021) 11:13264. 10.1038/s41598-021-92630-0 34168187 PMC8225908

[54] Sergi D Boulestin H Campbell FM Williams LM. The role of dietary advanced glycation end products in metabolic dysfunction. Mol Nutr Food Res. (2021) 65:e1900934. 10.1002/mnfr.201900934 32246887

[55] Sruthi CR Raghu KG. Advanced glycation end products and their adverse effects: the role of autophagy. J Biochem Mol Toxicol. (2021) 35:e22710. 10.1002/jbt.22710 33506967

[56] Sohouli MH Fatahi S Sharifi-Zahabi E Santos HO Tripathi N Lari A. The impact of low advanced glycation end products diet on metabolic risk factors: a systematic review and meta-analysis of randomized controlled trials. Adv Nutr. (2021) 12:766–76. 10.1093/advances/nmaa150 33253361 PMC8166565

[57] Walke PB Bansode SB More NP Chaurasiya AH Joshi RS Kulkarni MJ. Molecular investigation of glycated insulin-induced insulin resistance via insulin signaling and AGE-RAGE axis. Biochim Biophys Acta Mol Basis. (2021) 1867:166029. 10.1016/j.bbadis.2020.166029 33248275

[58] Manigrasso MB Juranek J Ramasamy R Schmidt AM. Unlocking the biology of RAGE in diabetic microvascular complications. Trends Endocrinol Metab. (2014) 25:15–22. 10.1016/j.tem.2013.08.002 24011512 PMC3877224

[59] El-Mesallamy HO Hamdy NM Ezzat OA Reda AM. Levels of soluble advanced glycation end product-receptors and other soluble serum markers as indicators of diabetic neuropathy in the foot. J Investig Med. (2011) 59:1233–8. 10.2310/JIM.0b013e318231db64 21941211

[60] Makita Z Radoff S Rayfield EJ Yang Z Skolnik E Delaney V. Advanced glycosylation end products in patients with diabetic nephropathy. N Engl J Med. (1991) 325:836–42. 10.1056/NEJM199109193251202 1875967

[61] Wang Y Zhong J Zhang X Liu Z Yang Y Gong Q. The role of HMGB1 in the pathogenesis of type 2 diabetes. J Diabetes Res. (2016) 2016: 2543268. 10.1155/2016/2543268 28101517 PMC5215175

[62] Yang H Wang H Czura CJ Tracey KJ. HMGB1 as a cytokine and therapeutic target. J Endotoxin Res. (2002) 8:469–72. 10.1179/096805102125001091 12697092

[63] Das N Dewan V Grace PM Gunn RJ Tamura R Tzarum N. HMGB1 activates proinflammatory signaling via TLR5 leading to allodynia. Cell Rep. (2016) 17:1128–40. 10.1016/j.celrep.2016.09.076 27760316 PMC5087801

[64] Ivanov S Dragoi AM Wang X Dallacosta C Louten J Musco G. A novel role for HMGB1 in TLR9-mediated inflammatory responses to CpG-DNA. Blood. (2007) 110:1970–81. 10.1182/blood-2006-09-044776 17548579 PMC1976374

[65] Yu M Wang H Ding A Golenbock DT Latz E Czura CJ. HMGB1 signals through toll-like receptor (TLR) 4 and TLR2. Shock. (2006) 26:174–9. 10.1097/01.shk.0000225404.51320.82 16878026

[66] Park JS Svetkauskaite D He Q Kim JY Strassheim D Ishizaka A. Involvement of toll-like receptors 2 and 4 in cellular activation by high mobility group box 1 protein. J Biol Chem. (2004) 279:7370–7. 10.1074/jbc.M306793200 14660645

[67] Behl T Sharma E Sehgal A Kaur I Kumar A Arora R. Expatiating the molecular approaches of HMGB1 in diabetes mellitus: highlighting signalling pathways via RAGE and TLRs. Mol Biol Rep. (2021) 48:1869–81. 10.1007/s11033-020-06130-x 33479829

[68] Nielsen TB Pantapalangkoor P Yan J Luna BM Dekitani K Bruhn K. Diabetes exacerbates infection via hyperinflammation by signaling through TLR4 and RAGE. mBio. (2017) 8:e00818–17. 10.1128/mBio.00818-17 28830942 PMC5565964

[69] Ferlita S Yegiazaryan A Noori N Lal G Nguyen T To K. Type 2 diabetes mellitus and altered immune system leading to susceptibility to pathogens, especially mycobacterium tuberculosis. J Clin Med. (2019) 8:2219. 10.3390/jcm8122219 31888124 PMC6947370

[70] Trinchieri G. Interleukin-12 and the regulation of innate resistance and adaptive immunity. Nat Rev Immunol. (2003) 3:133–46. 10.1038/nri1001 12563297

[71] Lagman M Ly J Saing T Kaur Singh M Vera Tudela E Morris D. Investigating the causes for decreased levels of glutathione in individuals with type II diabetes. PLoS ONE. (2015) 10:e0118436. 10.1371/journal.pone.0118436 25790445 PMC4366217

[72] Eckold C Kumar V Weiner J Alisjahbana B Riza AL Ronacher K. Impact of intermediate hyperglycemia and diabetes on immune dysfunction in tuberculosis. Clin Infect Dis. (2021) 72:69–78. 10.1093/cid/ciaa751 32533832 PMC7823074

[73] Crevel RV Critchley JA. The interaction of diabetes and tuberculosis: translating research to policy and practice. Trop Med Infect Dis. (2021) 6:8. 10.3390/tropicalmed6010008 33435609 PMC7838867

[74] Chávez-Reyes J Escárcega-González CE Chavira-Suárez E León-Buitimea A Vázquez-León P Morones-Ramírez JR. Susceptibility for some infectious diseases in patients with diabetes: the key role of glycemia. Front Public Health. (2021) 9:559595. 10.3389/fpubh.2021.559595 33665182 PMC7921169

[75] Hu R Xia CQ Butfiloski E Clare-Salzler M. Effect of high glucose on cytokine production by human peripheral blood immune cells and type I interferon signaling in monocytes: implications for the role of hyperglycemia in the diabetes inflammatory process and host defense against infection. Clin. Immunol. (2018) 195:139–48. 10.1016/j.clim.2018.06.003 29894743 PMC6119493

[76] Pedersen AML Sørensen CE Proctor GB Carpenter GH Ekström J. Salivary secretion in health and disease. J Oral Rehabil. (2018) 45:730–46. 10.1111/joor.12664 29878444

[77] López-Pintor RM Casañas E González-Serrano J Serrano J Ramírez L Arriba L. Xerostomia, hyposalivation, and salivary flow in Diabetes patients. J Diabetes Res. (2016) 2016:4372852. 10.1155/2016/4372852 27478847 PMC4958434

[78] Pappa E Vastardis H Rahiotis C. Chair-side saliva diagnostic tests: an evaluation tool for xerostomia and caries risk assessment in children with type 1 diabetes. J Dent. (2020) 93:103224. 10.1016/j.jdent.2019.103224 31722239

[79] Carda C Carranza M Arriaga A Diaz A Peydr,ó A Ferraris MEG. Structural differences between alcoholic and diabetic parotid sialosis. Med Oral Patol Oral Cir Bucal. (2005) 10:309–14. 16056184

[80] Negrato CA Tarzia O. Buccal alterations in diabetes mellitus. Diabetol Metab Syndr. (2010) 2:3. 10.1186/1758-5996-2-3 20180965 PMC2843640

[81] Kampoo K Teanpaisan R Ledder RG McBain AJ. Oral bacterial communities in individuals with type 2 diabetes who live in southern Thailand. Appl Environ Microbiol. (2014) 80:662–71. 10.1128/AEM.02821-13 24242241 PMC3911101

[82] Chen B Wang Z Wang J Su X Yang J Zhang Q. The oral microbiome profile and biomarker in Chinese type 2 diabetes mellitus patients. Endocrine. (2020) 68:564–72. 10.1007/s12020-020-02269-6 32246318

[83] Ogawa T Honda-Ogawa M Ikebe K Notomi Y Iwamoto Y Shirobayashi I. Characterizations of oral microbiota in elderly nursing home residents with diabetes. J Oral Sci. (2017) 59:549–55. 10.2334/josnusd.16-0722 28993578

[84] Goodson JM Hartman ML Shi P Hasturk H Yaskell T Vargas J. The salivary microbiome is altered in the presence of a high salivary glucose concentration. PLoS ONE. (2017) 12:e0170437. 10.1371/journal.pone.0170437 28249034 PMC5331956

[85] Ismail AF McGrath CP Yiu CKY. Oral health of children with type 1 diabetes mellitus: a systematic review. Diabetes Res Clin Pract. (2015) 108:369–81. 10.1016/j.diabres.2015.03.003 25817182

[86] Coelho AS Amaro IF Caramelo F Paula A Marto CM Ferreira MM. Dental caries, diabetes mellitus, metabolic control and diabetes duration: a systematic review and meta-analysis. J Esthet Restor Dent. (2020) 32:291–309. 10.1111/jerd.12562 31912978

[87] Twetman S Petersson GH Bratthall D. Caries risk assessment as a predictor of metabolic control in young type 1 diabetics. Diabet Med. (2005) 22:312–5. 10.1111/j.1464-5491.2005.01419.x 15717880

[88] Kogawa EM Grisi DC Falcao DP Amorim IA Rezende TMB Silva ICR. Impact of glycemic control on oral health status in type 2 diabetes individuals and its association with salivary and plasma levels of chromogranin A. Arch Oral Biol. (2016) 62:10–9. 10.1016/j.archoralbio.2015.11.005 26605682

[89] Malvania EA Sheth SA Sharma AS Mansuri S Shaikh F Sahani S. Dental caries prevalence among type II diabetic and nondiabetic adults attending a hospital. J Int Soc Prevent Commun Dent. (2016) 6:S232–6. 10.4103/2231-0762.197202 28217542 PMC5285600

[90] de Lima AKA Amorim SJ Stefani CM Almeida de Lima A Damé-Teixeira N. Diabetes mellitus and poor glycemic control increase the occurrence of coronal and root caries: a systematic review and meta-analysis. Clin Oral Investig. (2020) 24:3801–12. 10.1007/s00784-020-03531-x 32829477

[91] Li X Kolltveit KM Tronstad L Olsen I. Systemic diseases caused by oral infection. Clin Microbiol Rev. (2000) 13:547–58. 10.1128/CMR.13.4.547 11023956 PMC88948

[92] Taylor GW. Bidirectional interrelationships between diabetes and periodontal diseases: an epidemiologic perspective. Ann Periodontol. (2001) 6:99–112. 10.1902/annals.2001.6.1.99 11887478

[93] Lalla E Papapanou PN. Diabetes mellitus and periodontitis: a tale of two common interrelated diseases. Nat Rev Endocrinol. (2011) 7:738–48. 10.1038/nrendo.2011.106 21709707

[94] Chee B Park B Bartold PM. Periodontitis and type II diabetes: a two-way relationship. Int J Evid Based Health. (2013) 11:317–29. 10.1111/1744-1609.12038 24298927

[95] Polak D Sanui T Nishimura F Shapira L. Diabetes as a risk factor for periodontal disease-plausible mechanisms. Periodontol 2000. (2020) 83:46–58. 10.1111/prd.12298 32385872

[96] Minty M Canceil T Serino M Burcelin R Terc,é F Blasco-Baque V. Oral microbiota-induced periodontitis: a new risk factor of metabolic diseases. Rev Endocr Metab Disord. (2019) 20:449–59. 10.1007/s11154-019-09526-8 31741266

[97] Polak D Shapira L. An update on the evidence for pathogenic mechanisms that may link periodontitis and diabetes. J Clin Periodontol. (2018) 45:150–66. 10.1111/jcpe.12803 29280184

[98] Graziani F Gennai S Solini A Petrini M. A systematic review and meta-analysis of epidemiologic observational evidence on the effect of periodontitis on diabetes an update of the EFP-AAP review. J Clin Periodontol. (2018) 45:167–87. 10.1111/jcpe.12837 29277926

[99] Gaffen SL Hajishengallis G. A new inflammatory cytokine on the block: re-thinking periodontal disease and the Th1/Th2 paradigm in the context of Th17 cells and IL-17. J Dent Res. (2008) 87:817–28. 10.1177/154405910808700908 18719207 PMC2692983

[100] Moutsopoulos NM Konkel J Sarmadi M Eskan MA Wild T Dutzan N. Defective neutrophil recruitment in leukocyte adhesion deficiency type I disease causes local IL-17-driven inflammatory bone loss. Sci Transl Med. (2014) 6:229ra40. 10.1126/scitranslmed.3007696 24670684 PMC4090608

[101] Huang Z Pei X Graves DT. The interrelationship between diabetes, IL-17 and bone loss. Curr Osteoporos Rep. (2020) 18:23–31. 10.1007/s11914-020-00559-6 32002770 PMC10757470

[102] Gaffen SL Moutsopoulos NM. Regulation of host-microbe interactions at oral mucosal barriers by type 17 immunity. Sci Immunol. (2020) 5:eaau4594. 10.1126/sciimmunol.aau4594 31901072 PMC7068849

[103] Gárcia-Hernández A Arzate H Gil-Chavarría I Rojo R Moreno-Fierros L. High glucose concentrations alter the biomineralization process in human osteoblastic cells. Bone. (2012) 50:276–88. 10.1016/j.bone.2011.10.032 22086137

[104] Wong SK Chin KY Suhaimi FH Ahmad F Ima-Nirwana S. The relationship between metabolic syndrome and osteoporosis: a review. Nutrients. (2016) 8:347. 10.3390/nu8060347 27338453 PMC4924188

[105] Yamaguchi M. Role of nutritional zinc in the prevention of osteoporosis. Mol Cell Biochem. (2010) 338:241–54. 10.1007/s11010-009-0358-0 20035439

[106] Yuan Y Chen X Zhang L Wu J Guo J Zou D. The roles of exercise in bone remodeling and in prevention and treatment of osteoporosis. Prog Biophys Mol Biol. (2016) 122:122–30. 10.1016/j.pbiomolbio.2015.11.005 26657214

[107] Ferreira ECS Bortolin RH Freire-Neto FP Souza KSC Bezerra JF Ururahy MAG. Zinc supplementation reduces RANKL/OPG ratio and prevents bone architecture alterations in ovariectomized and type 1 diabetic rats. Nutr Res. (2017) 40:48–56. 10.1016/j.nutres.2017.03.004 28473060

[108] Lu R Zheng Z Yin Y Jiang Z. Genistein prevents bone loss in type 2 diabetic rats induced by streptozotocin. Food Nutr Res. (2020) 9:64. 10.29219/fnr.v64.3666 33447176 PMC7778425

[109] Silva VdO Lobato RV Andrade EF de Macedo CG Napimoga JT Napimoga MH. (2015) β-glucans (*Saccharomyces cereviseae*) reduce glucose levels and attenuate alveolar bone loss in diabetic rats with periodontal disease. PLoS ONE. 10:e0134742. 10.1371/journal.pone.0134742 26291983 PMC4546386

[110] de O Silva V Lobato RV Andrade EF Orlando DR Borges BDB Zangeronimo MG. Effects of β-glucans ingestion on alveolar bone loss, intestinal morphology, systemic inflammatory profile, and pancreatic β-cell function in rats with periodontitis and diabetes. Nutrients. (2017) 9:1016. 10.3390/nu9091016 28906456 PMC5622776

[111] Archana A Sasireka S Prabhu M Bobby B Srikanth V. Correlation between circulatory and salivary il 10 levels in periodontal health and disease-a report. Int J Dent Sci Res. (2014) 2:7–10. 10.12691/ijdsr-2-4B-3

[112] Andrade EF Orlando DR Gomes JAS Foureaux RC Costa RC Varaschin MS. Exercise attenuates alveolar bone loss and anxiety-like behaviour in rats with periodontitis. J Clin Periodontol. (2017) 44:1153–63. 10.1111/jcpe.12794 28800160

[113] Andrade EF Silva VO Moura NO Foureaux RC Orlando DR Moura RF. Physical exercise improves glycemic and inflammatory profile and attenuates progression of periodontitis in diabetic rats (HFD/STZ). Nutrients. (2018) 10:1702. 10.3390/nu10111702 30405072 PMC6265772

[114] Shi B Lux R Klokkevold P Chang M Barnard E Haake S. The subgingival microbiome associated with periodontitis in type 2 diabetes mellitus. ISME J. (2020) 14:519–530. 10.1038/s41396-019-0544-3 31673077 PMC6976570

[115] Matsha TE Prince Y Davids S Chikte U Erasmus RT Kengne AP. Oral microbiome signatures in diabetes mellitus and periodontal disease. J Dent Res. (2020) 99:658–65. 10.1177/0022034520913818 32298191

[116] Saeb ATM Al-Rubeaan KA Aldosary K Udaya Raja GK Mani B Abouelhoda M. Relative reduction of biological and phylogenetic diversity of the oral microbiota of diabetes and pre-diabetes patients. Microb Pathog. (2019) 128:215–29. 10.1016/j.micpath.2019.01.009 30625362

[117] Farina R Sever M Carrieri A Miotto E Sabbioni S Trombelli L. Whole metagenomic shotgun sequencing of the subgingival microbiome of diabetics and non-diabetics with different periodontal conditions. Arch Oral Biol. (2019) 104:13–23. 10.1016/j.archoralbio.2019.05.025 31153098

[118] Mealey BL Rose LF. Diabetes mellitus and inflammatory periodontal diseases. Curr Opin Endocrinol Diabetes Obes. (2008) 15:135–41. 10.1097/MED.0b013e3282f824b7 18316948

[119] D'Aiuto F Gkranias N Bhowruth D Khan T Orlandi M Suvan J. Systemic effects of periodontitis treatment in patients with type 2 diabetes: a 12 month, single-centre, investigator-masked, randomised trial. Lancet Diabetes Endocrinol. (2018) 6:954–65. 10.1016/S2213-8587(18)30038-X 30472992

[120] Hajishengallis G Chavakis T. Local and systemic mechanisms linking periodontal disease and inflammatory comorbidities. Nat Rev Immunol. (2021) 2021:1–5. 10.1038/s41577-020-00488-6 33510490 PMC7841384

[121] Chen YF Zhan Q Wu CZ Yuan H Chen W Yu FY. Baseline HbA1c level influences the effect of periodontal therapy on glycemic control in people with type 2 diabetes and periodontitis: a systematic review on randomized controlled trails. Diabetes Ther. (2021) 12:1249–78. 10.1007/s13300-021-01036-8 33481189 PMC8099950

[122] Jiang X Zhu Y Liu Z Tian Z Zhu S. Association between diabetes and dental implant complications: a systematic review and meta-analysis. Acta Odontol Scand. (2021) 79:9–18. 10.1080/00016357.2020.1761031 32401121

[123] Vasconcelos BCE Novaes M Sandrini FAL Maranhão Filho AWA Coimbra LS. Prevalence of oral mucosa lesions in diabetic patients: a preliminary study. Braz J Otorhinolaryngol. (2008) 74:423–8. 10.1016/S1808-8694(15)30578-4 18661018 PMC9442128

[124] Bastos AS Leite ARP Spin-Neto R Nassar PO Massucato EMS Orrico SRP. Diabetes mellitus and oral mucosa alterations: prevalence and risk factors. Diabetes Res Clin Pract. (2011) 92:100–5. 10.1016/j.diabres.2011.01.011 21300417

[125] González-Serrano J Serrano J López-Pintor RM Paredes VM Casañas E Hernández G. Prevalence of oral mucosal disorders in diabetes mellitus patients compared with a control group. J Diabetes Res. (2016) 2016:5048967. 10.1155/2016/5048967 27847829 PMC5099460

[126] Jhugroo C Divakar DD Jhugroo P Al-Amri SAS Alahmari AD Vijaykumar S. Characterization of oral mucosa lesions and prevalence of yeasts in diabetic patients: a comparative study. Microb Pathog. (2019) 126:363–7. 10.1016/j.micpath.2018.11.028 30471434

[127] Irfan M Delgado RZR Frias-Lopez J. The oral microbiome and cancer. Front Immunol. (2020) 11:591088. 10.3389/fimmu.2020.591088 33193429 PMC7645040

[128] Ramos-Garcia P Roca-Rodriguez MDM Aguilar-Diosdado M Gonzalez-Moles MA. Diabetes mellitus and oral cancer/oral potentially malignant disorders: a systematic review and meta-analysis. Oral Dis. (2020) 27:404–21. 10.1111/odi.13289 31994293

[129] Chilamakuri R Agarwal S. COVID-19: characteristics and therapeutics. Cells. (2021) 10:206. 10.3390/cells10020206 33494237 PMC7909801

[130] Choi HM Moon SY Yang HI Kim KS. Understanding viral infection mechanisms and patient symptoms for the development of COVID-19 therapeutics. Int J Mol Sci. (2021) 22:1737. 10.3390/ijms22041737 33572274 PMC7915126

[131] Siddiqi HK Mehra MR. COVID-19 illness in native and immunosuppressed states: a clinical-therapeutic staging proposal. J Heart Lung Transplant. (2020) 39:405–7. 10.1016/j.healun.2020.03.012 32362390 PMC7118652

[132] Santos A Magro DO Evangelista-Poderoso R Saad MJA. Diabetes, obesity, and insulin resistance in COVID-19: molecular interrelationship and therapeutic implications. Diabetol Metab Syndr. (2021) 13:23. 10.1186/s13098-021-00639-2 33648564 PMC7919999

[133] To KK Tsang OT Yip CC Chan KH Wu TC Chan JM. Consistent detection of 2019 novel coronavirus in saliva. Clin Infect Dis. (2020) 71:841–3. 10.1093/cid/ciaa149 32047895 PMC7108139

[134] Yoon JG Yoon J Song JY Yoon SY Lim CS Seong H. Clinical significance of a high SARS-CoV-2 viral load in the saliva. J Korean Med Sci. (2020) 35:e195. 10.3346/jkms.2020.35.e195 32449329 PMC7246183

[135] Carrouel F Gonçalves LS Conte MP Campus G Fisher J Fraticelli L. Antiviral activity of reagents in mouth rinses against SARS-CoV-2. J Dent Res. (2021) 100:124–32. 10.1177/0022034520967933 33089717 PMC7582358

[136] Kuba K Imai Y Rao S Gao H Guo F Guan B. A crucial role of angiotensin converting enzyme 2 (ACE2) in SARS coronavirus-induced lung injury. Nat Med. (2005) 11:875–9. 10.1038/nm1267 16007097 PMC7095783

[137] Liu L Wei Q Alvarez X Wang H Du Y Zhu H. Epithelial cells lining salivary gland ducts are early target cells of severe acute respiratory syndrome coronavirus infection in the upper respiratory tracts of Rhesus Macaques. J Virol. (2011) 85:4025–30. 10.1128/JVI.02292-10 21289121 PMC3126125

[138] Coke CJ Davison B Fields N Fletcher J Rollings J Roberson L. SARS-CoV-2 infection and oral health: therapeutic opportunities and challenges. J Clin Med. (2021) 10:156. 10.3390/jcm10010156 33466289 PMC7795434

[139] Pitones-Rubio V Chávez-Cortez EG Hurtado-Camarena A González-Rascón A Serafín-Higuera N. Is periodontal disease a risk factor for severe COVID-19 illness? Med Hypotheses. (2020) 144:109969. 10.1016/j.mehy.2020.109969 32592918 PMC7303044

[140] Xiang Z Koo H Chen Q Zhou X Liu Y Simon-Soro A. Potential implications of SARS-CoV-2 oral infection in the host microbiota. J Oral Microbiol. (2021) 13:1853451. 10.1080/20002297.2020.1853451 33312449 PMC7711743

[141] Bao L Zhang C Dong J Zhao L Li Y Sun J. Oral microbiome and SARS-CoV-2: beware of lung co-infection. Front Microbiol. (2020) 11:1840. 10.3389/fmicb.2020.01840 32849438 PMC7411080

[142] Corona G Pizzocaro A Vena W Rastrelli G Semeraro F Isidori AM. Diabetes is most important cause for mortality in COVID-19 hospitalized patients: systematic review and meta-analysis. Rev Endocr Metab Disord. (2021) 2021:1–22. 10.1007/s11154-021-09630-8 33616801 PMC7899074

[143] Apicella M Campopiano MC Mantuano M Mazoni L Coppelli A Del Prato. S. COVID-19 in people with diabetes: understanding the reasons for worse outcomes. Lancet Diabetes Endocrinol. (2020) 8:782–92. 10.1016/S2213-8587(20)30238-2 32687793 PMC7367664

[144] Eyth E Naik R. Hemoglobin A1C. Treasure Island, FL: StatPearls Publishing (2021). 31747223

[145] American Diabetes Association. Standards of medical care in diabetes-2016 abridged for primary care providers. Clin Diabetes. (2016) 34, 3–21. 10.2337/diaclin.34.1.3 26807004 PMC4714725

[146] Reynolds AN Akerman AP Mann J. Dietary fibre and whole grains in diabetes management: Systematic review and meta-analyses. PLoS Med. (2020) 17:e1003053. 10.1371/journal.pmed.1003053 32142510 PMC7059907

[147] Umpierrez GE Isaacs SD Bazargan N You X Thaler LM Kitabchi AE. Hyperglycemia: an independent marker of in-hospital mortality in patients with undiagnosed diabetes. J Clin Endocrinol Metab. (2002) 87:978–82. 10.1210/jcem.87.3.8341 11889147

[148] Yi H Huang J Guo L Zhang Q Qu J Zhou M. Increased antimicrobial resistance among sputum pathogens from patients with hyperglycemia. Infect Drug Resist. (2020) 13:1723–33. 10.2147/IDR.S243732 32606822 PMC7295332

[149] Satish BNVS Srikala P Maharudrappa B Awanti SM Kumar P. Saliva: a tool in assessing glucose levels in diabetes mellitus. J Int Oral Health. (2014) 6:114–7. 24876711 PMC4037799

[150] Carramolino-Cuéllar E Lauritano D Carinci F Silvestre-Rangil J Bañuls-Morant C Silvestre FJ. Salivary glucose as a metabolic control marker in patients with type 2 diabetes. J Biol Regul Homeost Agents. (2017) 31:181–7. 28691471

[151] Fares S Said MSM Ibrahim W Amin TT Saad NES. Accuracy of salivary glucose assessment in diagnosis of diabetes and prediabetes. Diabetes Metab Syndr. (2019) 13:1543–7. 10.1016/j.dsx.2019.03.010 31336519

[152] Tiongco REG Arceo ES Rivera NS Flake CCD Policarpio AR. Estimation of salivary glucose, amylase, calcium, and phosphorus among non-diabetics and diabetics: Potential identification of non-invasive diagnostic markers. Diabetes Metab Syndr. (2019) 13:2601–5. 10.1016/j.dsx.2019.07.037 31405682

[153] Mascarenhas P Fatela B Barahona I. Effect of diabetes mellitus type 2 on salivary glucose–a systematic review and meta-analysis of observational studies. PLoS ONE. (2014) 9:e101706. 10.1371/journal.pone.0101706 25025218 PMC4098915

[154] Miranda TS Feres M Retamal-Valdés B Perez-Chaparro PJ Maciel SS Duarte PM. Influence of glycemic control on the levels of subgingival periodontal pathogens in patients with generalized chronic periodontitis and type 2 diabetes. J Appl Oral Sci. (2017) 25:82–9. 10.1590/1678-77572016-0302 28198980 PMC5289404

[155] Komazaki R Katagiri S Takahashi H Maekawa S Shiba T Takeuchi Y. Periodontal pathogenic bacteria, *Aggregatibacter actinomycetemcomitans* affect non-alcoholic fatty liver disease by altering gut microbiota and glucose metabolismo. Sci Rep. (2017) 7:13950. 10.1038/s41598-017-14260-9 29066788 PMC5655179

[156] Todosenko N Vulf M Yurova K Skuratovskaia D Khaziakhmatova O Gazatova N. The pathogenic subpopulation of Th17 cells in obesity. Curr Pharm Des. (2021). 10.2174/1381612826666210101154913. [Epub ahead of print]. 33388015

[157] Li C Xu MM Wang K Adler AJ Vella AT Zhou B. Macrophage polarization and meta-inflammation. Transl Res. (2018) 191:29–44. 10.1016/j.trsl.2017.10.004 29154757 PMC5776711

[158] Tilg H Moschen AR. Adipocytokines: mediators linking adipose tissue, inflammation and immunity. Nat Rev Immunol. (2006) 6:772–83. 10.1038/nri1937 16998510

[159] Eljaafari A Robert M Chehimi M Chanon S Durand C Vial G. Adipose tissue-derived stem cells from obese subjects contribute to inflammation and reduced insulin response in adipocytes through differential regulation of the Th1/Th17 balance and monocyte activation. Diabetes. (2015) 64:2477–88. 10.2337/db15-0162 25765019

[160] Maximus PS Al Achkar Z Hamid PF Hasnain SS Peralta CA. Adipocytokines: are they the theory of everything? Cytokine. (2020) 133:155144. 10.1016/j.cyto.2020.155144 32559663 PMC7297161

[161] Havel PJ. Update on adipocyte hormones: regulation of energy balance and carbohydrate/lipid metabolism. Diabetes. (2004) 53:S143. 10.2337/diabetes.53.2007.S143 14749280

[162] Choi HM Doss HM Kim KS. Multifaceted physiological roles of adiponectin in Inflammation and diseases. Int J Mol Sci. (2020) 21:1219. 10.3390/ijms21041219 32059381 PMC7072842

[163] Chehimi M Robert M El Bechwaty M Vial G Rieusset J Vidal H. Adipocytes, like their progenitors, contribute to inflammation of adipose tissues through promotion of Th-17 cells and activation of monocytes, in obese subjects. Adipocyte. (2016) 5:275–82. 10.1080/21623945.2015.1134402 27617173 PMC5013985

[164] Fantuzzi G. Adipose tissue, adipokines, and inflammation. J Allergy Clin Immunol. (2005) 115:911–9. 10.1016/j.jaci.2005.02.023 15867843

[165] Varol C Mildner A Jung S. Macrophages: development and tissue specialization. Annu Rev Immunol. (2015) 33:643–75. 10.1146/annurev-immunol-032414-112220 25861979

[166] Russo L Lumeng CN. Properties and functions of adipose tissue macrophages in obesity. Immunology. (2018) 155:407–17. 10.1111/imm.13002 30229891 PMC6230999

[167] Lumeng CN Bodzin JL Saltiel AR. Obesity induces a phenotypic switch in adipose tissue macrophage polarization. J Clin Invest. (2007) 117:175–84. 10.1172/JCI29881 17200717 PMC1716210

[168] Xu H Barnes GT Yang Q Tan G Yang D Chou CJ. Chronic inflammation in fat plays a crucial role in the development of obesity-related insulin resistance. J Clin Invest. (2003) 112:1821–30. 10.1172/JCI200319451 14679177 PMC296998

[169] Wen J Liu Q Liu M Wang B Li M Wang M. Increasing imbalance of Treg/Th17 indicates more severe glucose metabolism dysfunction in overweight/obese patients. Arch Med Res. (2020) 52:339–47. 10.1016/j.arcmed.2020.11.012 33317842

[170] Wang M Chen F Wang J Zeng Z Yang Q Shao S. Th17 and Treg lymphocytes in obesity and Type 2 diabetic patients. Clin Immunol. (2018) 197:77–85. 10.1016/j.clim.2018.09.005 30218707

[171] Feuerer M Herrero L Cipolletta D Naaz A Wong J Nayer A. Lean, but not obese, fat is enriched for a unique population of regulatory T cells that affect metabolic parameters. Nat Med. (2009) 15:930–9. 10.1038/nm.2002 19633656 PMC3115752

[172] Jagannathan-Bogdan M McDonnell ME Shin H Rehman Q Hasturk H Apovian CM. Elevated proinflammatory cytokine production by a skewed T cell compartment requires monocytes and promotes inflammation in type 2 diabetes. J Immunol. (2011) 186:1162–72. 10.4049/jimmunol.1002615 21169542 PMC3089774

[173] Bettelli E Carrier Y Gao W Korn T Strom TB Oukka M. Reciprocal developmental pathways for the generation of pathogenic effector TH17 and regulatory T cells. Nature. (2006) 441:235–8. 10.1038/nature04753 16648838

[174] Acosta-Rodriguez EV Napolitani G Lanzavecchia A Sallusto F. Interleukins 1beta and 6 but not transforming growth factor-beta are essential for the differentiation of interleukin 17-producing human T helper cells. Nat Immunol. (2007) 8:942–9. 10.1038/ni1496 17676045

[175] Abdel-Moneim A Bakery HH Allam G. The potential pathogenic role of IL-17/Th17 cells in both type 1 and type 2 diabetes mellitus. Biomed Pharmacother. (2018) 101:287–92. 10.1016/j.biopha.2018.02.103 29499402

[176] Zheng Z Zheng F. A complex auxiliary: IL-17/Th17 signaling during type 1 diabetes progression. Mol Immunol. (2019) 105:16–31. 10.1016/j.molimm.2018.11.007 30472513

[177] Goodson JM Groppo D Halem S Carpino E. Is obesity an oral bacterial disease? J Dent Res. (2009) 88:519–23. 10.1177/0022034509338353 19587155 PMC2744897

[178] Tam J Hoffmann T Fischer S Bornstein S Gräßler J Noack B. Obesity alters composition and diversity of the oral microbiota in patients with type 2 diabetes mellitus independently of glycemic control. PLoS ONE. (2018) 13:e0204724. 10.1371/journal.pone.0204724 30273364 PMC6166950

[179] Janem WF Scannapieco FA Sabharwal A Tsompana M Berman HA Haase EM. Salivary inflammatory markers and microbiome in normoglycemic lean and obese children compared to obese children with type 2 diabetes. PLoS ONE. (2017) 12:e0172647. 10.1371/journal.pone.0172647 28253297 PMC5333807

[180] Araujo DS Klein MI Scudine KGO Leite LS Parisotto TM Ferreira CM. Salivary microbiological and gingival health status evaluation of adolescents with overweight and obesity: a cluster analysis. Front Pediatr. (2020) 8:429. 10.3389/fped.2020.00429 32850543 PMC7411150

[181] Iqbal AM Jamal SF. Essential Hypertension. Treasure Island, FL: StatPearls Publishing (2020). 30969681

[182] Aronow WS Shamliyan TA. Blood pressure targets for hypertension in patients with type 2 diabetes. Ann Transl Med. (2018) 6:199. 10.21037/atm.2018.04.36 30023362 PMC6035980

[183] Paizan MLM Vilela-Martin JF. Is there an association between periodontitis and hypertension? Curr Cardiol Rev. (2014) 10:355–61. 10.2174/1573403X10666140416094901 24739001 PMC4101200

[184] Vidal F Cordovil I Figueredo CMS Fischer RG. Non-surgical periodontal treatment reduces cardiovascular risk in refractory hypertensive patients: a pilot study. J Clin Periodontol. (2013) 40:681–7. 10.1111/jcpe.12110 23639076

[185] Pietropaoli D Del Pinto R Ferri C Ortu E Monaco A. Definition of hypertension-associated oral pathogens in NHANES. J Periodontol. (2019) 90:866–76. 10.1002/JPER.19-0046 31090063

[186] Pietropaoli D Monaco A D'Aiuto F Aguilera EM Ortu E Giannoni M. Active gingival inflammation is linked to hypertension. J Hypertens. (2020) 38:2018–27. 10.1097/HJH.0000000000002514 32890278

[187] Czesnikiewicz-Guzik M Nosalski R Mikolajczyk TP Vidler F Dohnal T Dembowska E. Th1-type immune responses to *Porphyromonas gingivalis* antigens exacerbate angiotensin II-dependent hypertension and vascular dysfunction. Br J Pharmacol. (2019) 176:1922–31. 10.1111/bph.14536 30414380 PMC6534780

[188] Oliveira-Paula GH Pinheiro LC Tanus-Santos JE. Mechanisms impairing blood pressure responses to nitrite and nitrate. Nitric Oxide. (2019) 85:35–43. 10.1016/j.niox.2019.01.015 30716418

[189] Hezel MP Weitzberg E. The oral microbiome and nitric oxide homoeostasis. Oral Dis. (2015) 21:7–16. 10.1111/odi.12157 23837897

[190] Lundberg JO Weitzberg E Cole JA Benjamin N. Nitrate, bacteria and human health. Nat Rev Microbiol. (2004) 2:593–602. 10.1038/nrmicro929 15197394

[191] Lundberg JO Weitzberg E Gladwin MT. The nitrate-nitrite-nitric oxide pathway in physiology and therapeutics. Nat Rev Drug Discov. (2008) 7:156–67. 10.1038/nrd2466 18167491

[192] Vanhatalo A Blackwell JR L'Heureux JE Williams DW Smith A van der Giezen M. Nitrate-responsive oral microbiome modulates nitric oxide homeostasis and blood pressure in humans. Free Radic Biol Med. (2018) 124:21–30. 10.1016/j.freeradbiomed.2018.05.078 29807159 PMC6191927

[193] Hyde ER Luk B Cron S Kusic L McCue T Bauch T. Characterization of the rat oral microbiome and the effects of dietary nitrate. Free Radic Biol Med. (2014) 77:249–57. 10.1016/j.freeradbiomed.2014.09.017 25305639

[194] Burleigh MC Liddle L Monaghan C Muggeridge DJ Sculthorpe N Butcher JP. Salivary nitrite production is elevated in individuals with a higher abundance of oral nitrate-reducing bacteria. Free Radic Biol Med. (2018) 120:80–8. 10.1016/j.freeradbiomed.2018.03.023 29550328

[195] Velmurugan S Gan JM Rathod KS Khambata RS Ghosh SM Hartley A. Dietary nitrate improves vascular function in patients with hypercholesterolemia: a randomized, double-blind, placebo-controlled study. Am J Clin Nutr. (2016) 103:25–38. 10.3945/ajcn.115.116244 26607938 PMC4691670

[196] Bryan NS Tribble G Angelov N. Oral microbiome and nitric oxide: the missing link in the management of blood pressure. Curr Hypertens Rep. (2017) 19:33. 10.1007/s11906-017-0725-2 28353075

[197] Blekkenhorst LC Bondonno NP Liu AH Ward NC Prince R Lewis JR. Nitrate, the oral microbiome, and cardiovascular health: a systematic literature review of human and animal studies. Am J Clin Nutr. (2018) 107:504–22. 10.1093/ajcn/nqx046 29635489

[198] Ghasemi A Jeddi S. Anti-obesity and anti-diabetic effects of nitrate and nitrite. Nitric Oxide. (2017) 70:9–24. 10.1016/j.niox.2017.08.003 28804022

[199] Mooradian AD. Dyslipidemia in type 2 diabetes mellitus. Nat Clin Pract Endocrinol Metab. (2009) 5:150–9. 10.1038/ncpendmet1066 19229235

[200] Lazarte J Hegele RA. Dyslipidemia management in adults with diabetes. Can J Diabetes. (2020) 44:53–60. 10.1016/j.jcjd.2019.07.003 31521544

[201] Rivera MF Lee JY Aneja M Goswami V Liu L Velsko IM. Polymicrobial infection with major periodontal pathogens induced periodontal disease and aortic atherosclerosis in hyperlipidemic ApoE(null) mice. PLoS ONE. (2013) 8:e57178. 10.1371/journal.pone.0057178 23451182 PMC3581444

[202] Chukkapalli SS Velsko IM Rivera-Kweh MF Zheng D Lucas AR Kesavalu L. Polymicrobial oral infection with four periodontal bacteria orchestrates a distinct inflammatory response and atherosclerosis in ApoE null mice. PLoS ONE. (2015) 10:e0143291. 10.1371/journal.pone.0143291 26619277 PMC4664240

[203] Choi Y-H Kosaka T Ojima M Sekine S Kokubo Y Watanabe M. Relationship between the burden of major periodontal bacteria and serum lipid profile in a cross-sectional Japanese study. BMC Oral Health. (2018) 18:77. 10.1186/s12903-018-0536-0 29728099 PMC5935931

[204] Gaetti-Jardim E Marcelino SL Feitosa ACR Romito GA Avila-Campos MJ. Quantitative detection of periodontopathic bacteria in atherosclerotic plaques from coronary arteries. J Med Microbiol. (2009) 58:1568–75. 10.1099/jmm.0.013383-0 19679682

[205] Velsko IM Chukkapalli SS Rivera MF Lee J-Y Chen H Zheng D. Active invasion of oral and aortic tissues by *Porphyromonas gingivalis* in mice causally links periodontitis and atherosclerosis. PLoS ONE. (2014) 9:e97811. 10.1371/journal.pone.0097811 24836175 PMC4024021

[206] Køllgaard T Enevold C Bendtzen K Hansen PR Givskov M Holmstrup P. Cholesterol crystals enhance TLR2- and TLR4-mediated pro-inflammatory cytokine responses of monocytes to the proatherogenic oral bacterium *Porphyromonas gingivalis*. PLoS ONE. (2017) 12:e0172773. 10.1371/journal.pone.0172773 28235036 PMC5325525

[207] Kesavalu L Lucas AR Verma RK Liu L Dai E Sampson E. Increased atherogenesis during *Streptococcus mutans* infection in ApoE-null Mice. J Dent Res. (2012) 91:255–60. 10.1177/0022034511435101 22262633 PMC3275337

