# Supplementary material for: Interplay Among the Oral Microbiome, Oral Cavity Conditions, the Host Immune Response, Diabetes Mellitus, and Its Associated-Risk Factors—An Overview
Source: Front Oral Health. 2021 Sep 9;2:697428. doi: 10.3389/froh.2021.697428 (PMC8757730; doi:10.3389/froh.2021.697428)
Supplement: Supplementary file 1 [file Data_Sheet_1.DOCX]

Supplementary Material

* General features of immune response

In general, the host interacts with microbial cells through fine and complex responses regulated by mechanisms of innate and adaptive immunity. Innate immunity is classically defined as the host's first line of defense against microbial cells in an effort to maintain homeostasis and prevent the development of disease (Romo et al., 2017). From the earliest moments of microbial host-cell interactions, innate immune cells, such as phagocytic cells, and humoral factors, such as the complement system, are activated triggering the development of an inflammatory reaction in an effort to eliminate the pathogen (de Nardo, 2017; Netea et al., 2019).

Innate immune cells are the first ones to respond against invading microorganisms. These cells, being represented by neutrophils and macrophages, express surface pattern recognition receptors (PRRs) that are able to recognize specific molecular patterns associated with pathogens (PAMPs). One of the main PRRs responsible for this recognition is the “Toll-like” type (TLR) receptors, being, to date, 10 receptors identified in humans. After the recognition of PAMPs, TLRs activate specific intracellular pathways, triggering a response to the aggressor microorganism (Akira et al., 2006; Kumar et al; 2011). The recognition of microorganisms by the innate immune system provides signals for the activation of the adaptive immune system. Specifically, TLR-4 recognizes lipopolysaccharides (LPS) that are glycolipids present in the outer membrane of Gram-negative bacteria. LPS molecules, also known as bacterial endotoxins, trigger the activation of cells in the immune system by increasing the production of inflammatory mediators (Lassenius et al., 2011). The correct activation of TLR4 is necessary for the initiation of the inflammatory process and elimination of microorganisms.

CD4 T helper (Th) cells play a key role in orchestrating adaptive immune responses to a wide variety of infections. Th cells are also involved in the pathogenesis of diverse inflammatory diseases. These cells help B cells in the production of antibodies and in the recruitment and activation of other immune cells, such as, macrophages, mast cells, neutrophils, eosinophils and basophils. In addition, Th cells also act directly on host tissue cells (epithelial and mucosal cells) during the process of immune response against pathogens (Zhu and Paul, 2010). Based on their functions, their pattern of cytokine secretion and their expression of specific transcription factors, Th cells are classified into Th1, Th2, Th9, Th17, Th22 (Mosmann et al. 1986; Mosmann and Coffman 1989; Paul and Seder 1994; Moseley et al., 2003; Zhu and Paul; 2010; Basu et al., 2012; Li et al., 2017; Zhu, 2018) and regulatory T cells (Treg). Treg cells are essential for regulating the magnitude of immune responses (Sakaguchi et al., 2010; Zhu, 2018), playing a crucial role in health and in disease due to their immunosuppressive properties against various cells of the immune system (Han et al., 2019). The microbiome plays a critical role in the modulation and in the development of the main components of the host's innate and adaptive immune system, while the immune system orchestrates the maintenance of the main characteristics of host-microbe symbiosis. In a genetically susceptible host, it is believed that imbalances in microbiota-immunity interactions under defined environmental contexts contribute to the pathogenesis of a multitude of immune-mediated diseases, but it is still unclear how the environmental factors act in transitioning a commensal microbiota to a pathogenic one (Zheng et al., 2020).

As already mentioned, commensal microbial cells tend to live in symbiosis with the host. The breakdown of dynamic and friendly interactions between the host and commensal microbial cells can lead to chronic inflammatory disorders, including autoimmunity, allergies and metabolic syndromes (Crump and Sahingur, 2016; Belkaid and Harrison, 2017). The host's first barriers against microbial cells are intact skin, mucosal surfaces and their secretion products, which are able to respond to microbial stimuli in a specific way that depends on both the tissue being threatened and the type of microbial cell. Therefore, a wide variety of responses may be needed to control each type of infection (Turvey and Broide, 2010). That means a coordinated set of immune responses against microbial cells and innocuous antigens is extremely important for maintaining host survival and immunity balance. In this context, the immune system can induce or contract its response, preserving the physiological and functional needs of each tissue (Belkaid and Harrison, 2017). There is an established balance between the microbiota and the host's immune responses (Rijkschroeff et al., 2018). This explains the maintenance of a health-related condition even in sites heavily colonized by microorganisms, such as the oral cavity, the gastrointestinal tract, and urinary tract and other body sites.

* Immune response in the oral cavity

In the oral cavity, the host's innate and adaptive immune responses are played by components derived from saliva and from mucosal cells exerting protective roles against infections and tissue damage.

Saliva plays an important role in maintaining oral health (Pedersen and Belstrom, 2019). In addition to allow the clearance of microorganisms, metabolites and debris from the oral cavity and to control the pH by means of its buffering molecules, saliva has many proteins and natural enzymes, such as mucins, statherin, cystatins, histatins, immunoglobulins (sIgA), lysozyme, peroxidase system and lactoferrins which exert antibacterial and antifungal effects (Pedersen and Belstrom, 2019). In addition to these components, antimicrobial peptides (also known as host defense peptides) produced by both epithelial cells and by leukocytes are part of the innate immune defense mechanisms (Khurshid et al. , 2018; Sztukowska et al., 2019).

Cellular blood constituents (mainly neutrophils, but also monocytes, lymphocytes and erythrocytes) reach the oral cavity via mucosal transudate, via intraoral bleeding or via crevicular gingival fluid (CGF) (Fábián et al., 2012; Bostanci and Belibasakis, 2018). Neutrophils are the main leukocyte cells present in CGF from both healthy and inflamed periodontal sites. The CGF also contains epithelial cells exfoliated from the junctional or sulcular epithelium being this exfoliation process accelerated during an inflammatory response (Bostanci and Belibasakis, 2018). All of these components contribute to the immune response elicited in the oral cavity (Taylor and Preshaw, 2016).

* Xerostomia and Hyposalivation

The normal stimulated salivary flow ranges from 0.7–2.0 mL / min, while the range of unstimulated salivary flow is around 0.3–0.4 mL / min. Hyposalivation (a common signal of salivary gland hypofunction) is diagnosed when the salivary flow rate is below the levels showed above. On the other hand, xerostomia is defined as a subjective and self-reported complain of dry mouth. It is important to mention that xerostomia may or may not be associated with hyposalivation. In some cases, xerostomia is a secondary symptom associated to qualitative and / or quantitative changes in saliva composition (Fox et al., 1985; Humphrey and Williamson, 2001; Villa et al., 2015). Overall, long term use of prescription drugs, such as anticoagulants, antidepressants, antihypertensives, antiretrovirals, hypoglycemics, levothyroxine, multivitamins and supplements, non-steroidal anti-inflammatory and steroid inhalers, head and neck radiotherapy and Sjögren's syndrome are among the most frequent causes of hyposalivation (Villa et al., 2015).

Hyposalivation has also been associated with the aging process. In order to determine the prevalence of hyposalivation in the elderly (≥ 60 years), Pina et al., (2020) screened studies published until February 2019. The analysis of thirteen included studies representing 3,885 individuals showed 33.37% of hyposalivation prevalence. In a systematic review carried out by Agostini et al. (2018) the estimated general prevalence of xerostomia is 22.0%, being this estimative greater in elderly (27.2%), but the authors warned about the high heterogeneity of the assessed studies. Xerostomia may also be induced as a side-effect of chronic use of prescription drugs as showed by a systematic review and meta-analysis carried out by Tan et al. (2018). The risk of xerostomia in elderly (aged ≥60) was higher in those making long-term use of prescription drugs for urinary incontinence, followed by antidepressants and by psycholeptics (Tan et al., 2018). Salivary hypofunction is associated with the occurrence of dysphonia, dysphagia, dysgeusia, burning mouth, dental caries and oral infectious diseases (Saleh et al., 2015). Some evidences suggest that the implementation of oral health promotion programs based on mouth exercises (for stretching of the lip, the lip corner, the tongue and the cheek aiming at promoting the strengthening of the masticatory muscle of the tongue, of the platysma muscle and of the masticatory muscle, respectively), as well as on speaking and swallowing exercises, frequent tooth brushing and salivary gland massage in individuals presenting salivary hypofunction significantly increase salivary secretion rates and oral health-related quality of life (Cho et al., 2012; Kim et al., 2019; Seo and Kim, 2020).

* Dental caries

Dental caries is a dynamic, non-communicable and multifactorial disease that results in mineral loss of dental hard tissues. It is mediated by the presence of undisturbed dental biofilm and modulated by diet, being also mediated by biological, behavior, psychosocial and environmental features (Machiulskiene et al., 2020). Environmental acidification caused by bacterial fermentation of carbohydrates and the levels of antimicrobial proteins, including components of innate and acquired immune systems, have direct effect on phenotypic and genotypic changes in the oral microbiota and in biofilm accumulation during caries progression (Takahashi and Nyvad, 2011; Zhang et al., 2018).

The earliest colonizers of the tooth surface are commensal streptococci, such as Streptococcus mitis (*S.mitis*), *Streptococcus sanguinis* (*S.sanguinis*), *Streptococcus gordonii* (*S.gordonii*) and other related taxa. Upon biofilm accumulation and increased frequency of carbohydrates exposure, the resulted acidic and anaerobic conditions select acidogenic and acid-tolerant microorganisms such as *Streptococcus mutans* (*S.mutans*), *Lactobacillus* spp., and others (Baker and Edlund, 2019). An increased abundance of Proteobacteria and Bacteroidetes, as well as *Neisseria* spp and *Porphyromonas* spp presented increased abundance in saliva of children affected by severe early childhood caries, whereas Firmicutes, Bacteroidetes, *Neisseria* spp, *Prevotella* spp, *S. mutans*, *Bifidobacterium* spp and *Scardovia* spp. were found in high abundance in dental cavities (Hurley et al., 2019). *Candida* spp. have also been frequently associated with aciduric bacteria, such as *S. mutans*, certain *Lactobacillus* spp and *Scardovia* spp. in cases of severe early childhood caries (Xiao et al., 2018).

* Periodontal diseases

Periodontal disease is a well-known oral condition frequently associated to the pathogenesis of several systemic diseases, including DM (Li et al., 2000). Periodontal disease is a pathological process that involves the periodontium, a term used to describe the supporting tissues of the teeth which include gums, alveolar bone, cementum and periodontal ligament. Gingivitis, found in up to 90% of the population, is a condition developed due to the accumulation of microorganisms and debris between the gum line and the tooth. The inflammatory state associated with gingivitis is reversible as long as there is an improvement in oral hygiene. Periodontitis, on the other hand, occurs when inflammation progresses beyond gingivitis to a state of chronic inflammatory disease. Under this condition, the microorganisms found on biofilms associated with gingivitis are able to colonize the subgingival pocket triggering an immune response against these invading microorganisms. However, the production of immune response mediators ultimately leads to the aggravation of the inflammatory response and to a destruction of the periodontium. Therefore, periodontitis leads to the loss of insertion of the periodontium and to alveolar bone loss which could result in tooth loss (Gasner and Schure, 2021).

Shifts in the microbial composition of the subgingival biofilm and exacerbated inflammation have been associated with the development and progression of periodontitis (Van Dyke et al., 2020). However, it is not clear if the microbial dysbiosis in periodontitis is responsible to initiate the disease or if dysbiosis is a consequence of disease initiation by excessive host inflammatory and immune responses. Periodontal pockets become an anaerobic environment full of breakdown products and proteins from tissues and blood which favor the growth of Gram-negative and proteolytic bacteria (Marsh and Zaura, 2017; Van Dyke et al., 2020). Meuric et al. (2017) have shown that the prevalence of *Veilonella* spp, *Neisseria* spp, *Rothia* spp, *Corynebacterium* spp and *Actinomyces* spp was higher in subgingival biofilms from healthy patients whereas the prevalence of *Eubacterium* ssp, *Campylobacter* ssp, *Treponema* ssp and *Tanerella* spp were higher in subgingival samples from chronic periodontitis patients. Microbiome analysis was correlated with the pocket depth showing specific signatures for microbial dysbiosis. Products from polyamine, butyric acid metabolism and arginine, proline and lysine degradation were identified in samples of patients with high periodontal inflammatory status showing that some metabolic bioproducts can be also be considered as biomarkers of periodontal dysbiosis (Sakanaka et al., 2017).

* Halitosis

Halitosis is a common problem that manifests as an unpleasant odor coming out of the oral cavity. It affects from 15% to 60% of the population and is one of the most frequent complaints from patients seeking dental care, being behind the complaints for dental caries and for periodontal disease. Halitosis may have an intra-oral or an extra-oral cause and it results both from pathological and from non-pathological conditions. The offensive breath odor has negative impact on social interactions and on quality of life of the affected patient (Loesche and Kazor, 2002; Aylıkcı and Çolak, 2013; Suzuki et al., 2019; Hampelska et al., 2020; Wu et al., 2020).

Although halitosis has multifactorial origins, approximately 90% of halitosis cases are associated with intra-oral causes (Suzuki et al., 2019). The most frequent factor associated with halitosis includes metabolic activity of microbial reservoirs found in the dorsum of the tongue and in the periodontal pockets of patients presenting periodontal disease. Other factors that increase the likelihood of halitosis include food impaction, poor oral hygiene, long-lasting tongue coat, uncleaned dentures, throat infections, dry mouth, smoking habit, alcohol consumption, eating habits, gastrointestinal and hepatic diseases, obesity, dehydration, long-lasting fasting periods, great physical effort, advanced age, stress and diabetes (Tangerman and Winkel, 2010; Hampelska et al., 2020, Wu et al., 2020).

Volatile sulfur compounds (VSCs), aromatic compounds, amines, short-chain or organic fatty acids, alcohols, aliphatic compounds, aldehydes and ketones are within the odoriferous substances associated with halitosis (Hampelska et al., 2020). The VSCs, such as hydrogen sulfide (H2S) and methyl mercaptan (CH3SH), are the main components of oral malodor. Gram-negative anaerobic bacteria metabolize sulfur-containing amino acids and VSCs are the end products of this proteolytic process. Gram-positive bacteria, on the other hand, also contributes to the production of VSCs since they cleave the sugar chains of glycoproteins and, thus, provide proteins to be metabolized by Gram-negative microorganisms (Suzuki et al., 2019). *Actinomyces* spp., *Bacteroides* spp., *Campylobacter rectus*, *Capnocytophaga* spp, *Centipeda* spp, *Dialister* spp., *Eubacterium* spp., *Eikella* spp, *Fusobacterium* spp., *Leptotrichia* spp., *Peptostreptococcus* spp., *Porphyromonas* spp., *Prevotella* spp., *Selenomonas* spp., *Solobacterium* spp., *Tannerella forsythia*, *Treponema denticola* and *Veillonella* spp are the oral bacteria most related to the synthesis of VSCs (Persson et al., 1990; Nakano et al., 2002; Amou et al., 2014; Suzuki et al., 2019; Hampelska et al., 2020).

Besides VSCs produced by the oral microbiota, other volatile compounds (not associated with microbial metabolism) exhaled by the body may also contribute to halitosis and they serve as diagnostic tool for certain diseases. Volatile organic compounds (VOCs) composed of nitrogen, oxygen, carbon dioxide, water and inert gases are exhaled during breathing and are also present in saliva, blood, human milk, skin secretions, urine and feces (Mazzone et al., 2008; Costello et al., 2014). Exhaled breath analysis can provide a simple diagnosis for various diseases, since it is non-invasive and painless for patients. Since antiquity (∼400 years BC), physicians have used exhaled VOCs as a yield in the evaluation of their patients (Phillips, 1992; Nakhleh et al., 2017). Breath concentrations of some VOCs may be associated with a certain disease conditions, such as the presence of high levels of acetone in diabetic patients (Choi et al., 2014) which is responsible for the characteristic fruity odor coming out of mouth of these patients (Shirasu and Touhara, 2011).

Ketone bodies are products of the catabolism of fatty-compounds. Under low carbohydrate intake and under excess of free circulating fatty acids, the latter are used as alternative substrates to glucose. Increased concentration of ketone bodies occur due to insufficient insulin secretion which compromises the use of glucose as an energy source (termed as ketoacidosis), due to prolonged periods of fasting (termed as mild ketonemia) or due to the consumption of carbohydrate-restricted diet (termed as ketogenic diet). The main components of ketone bodies are β-hydroxybutyrate (3-hydroxybutyrate; β-HB), acetoacetate (AcAc) and acetone. Under normal physiological conditions, ketone plasma levels range between 100 and 600 µM. In the presence of ketoacidosis, a severe acute complication of uncontrolled diabetes, ketone plasma levels increase to 1–1.5 mM (Laffel, 1999; Garcia et al., 2020). In this context, exhaled breath air has been used in some studies as a tool for monitoring DM. Garcia et al. (2020) showed that the levels of ketone bodies on plasma are elevated in DM2 individuals compared with non-diabetic individuals. Moreover, total ketone bodies and β-HB levels were directly associated with free fatty acids concentration and DM2 condition. Trefz et al. (2019) showed that the concentration of exhaled VOCs differed between patients with DM1 and healthy controls. DM1 patients exhaled significantly greater amounts of ethanol, isopropanol, dimethyl sulfide, isoprene and pentanal compared to healthy controls. The most notable differences in concentrations were found in patients with poor metabolic control, that is, those with an average HbA1c above 8%. Moreover, the presence of ethanol and acetone in the exhaled air has also been tested to estimate glucose blood levels. Whereas acetone is derived from the oxidation of free fatty acids which is influenced by glucose metabolism (as described above), ethanol is produced by the alcoholic fermentation of glucose by intestinal bacteria and yeasts. By analyzing the exhaled air and glucose blood levels of healthy individuals during an oral glucose tolerance test (OGTT) (ingestion of 75 g of glucose followed by 120 minutes of sampling), Galassetti et al. (2005) showed that the mean glucose blood levels exhibited a typical OGTT pattern (rapid increase, peak values in 30-60 min and gradual return to baseline in 120 min). Breathed ethanol showed a similar pattern (with peak values in 30 minutes and a rapid return to baseline levels at 60 min) whereas acetone levels decreased progressively below baseline levels, with lower readings obtained at 120 min. This way, authors showed that the integrated analysis of exhaled ethanol and acetone and glucose metabolism correlated with changes in glucose blood levels. Exhaled VOCs has now attracted considerable scientific and clinical interest as promissing biomarkers for the diagnosis of several diseases, including DM although methodological standardization is still demanded in order to allow a proper translation of these approaches to the clinical rotine (Shirasu and Touhara, 2011; Rondanelli et al., 2019; Dharmawardana et al., 2020, Peel et al., 2020).

* Interplay between oral microbiome, taste perception, smell recognition and DM

Feng et al. (2018) characterized the composition of saliva and the tongue coating and their main substrates and metabolic products (sugars and organic acids) in order to assess whether the microbial composition play a role in the sensitivity to the five basic flavors: sweet (fructose), salty (sodium chloride), acid (citric acid), bitter (quinine hydrochloride) and umami (monosodium glutamate). The results showed that the pH of saliva varied from 5.89 to 7.00 (mean 6.47 ± 0.29) and it was significantly lower than the pH of tongue film which varied from 6.53 to 7.86 (mean 7.15 ± 0.36). Of the four sugars (glucose, fructose, sucrose and lactose) and four organic acids (lactic acid, acetic acid, propionic acid and butyric acid), glucose and acetic acid were the most predominant sugars and acids in both saliva and tongue film. The correlation between sensitivity to taste and concentrations of organic acids (lactic, butyric and acetic) was stronger in saliva than in the tongue film and clearly indicates that higher salivary levels of organic acids are associated with greater sensitivity to acid taste. So far, the mechanism that links organic acids and taste sensitivity remains unknown. In contrast to organic acids, the correlation between sensitivity to sugar taste was stronger in the tongue film of the tongue than in saliva. Sucrose and lactose tended to be positively associated with taste sensitivity, while fructose and glucose monosaccharides were negatively associated with taste sensitivity. A higher level of sucrose in the tongue film may indicate a lower proportion of bacteria capable of converting them to glucan and fructan (or a lower conversion rate) and, therefore, a less tightly structured biofilm. The physical barrier between the taste and taste receptors would, as a consequence, is less efficient and the sensitivity increased. A more precise characterization of the bacterial genera and species in the tongue film and a targeted study of their sugar metabolism would be necessary to test this hypothesis. In relation to microbiological data, compared to saliva, the lingual film was characterized by significantly lower proportions of Bacteroidetes and its main genus *Prevotella* spp and significantly higher proportions of Firmicutes, Actinobacteria, and the genus *Streptococcus* spp. It is suggested that higher proportions of Actinobacteria and Firmicutes in saliva are linked to lower taste sensitivity, while a higher proportion of Bacteroidetes in the tongue film increases sensitivity (particularly to bitterness). Although more detailed characterizations of microbial communities and their metabolism are necessary, this study, despite its limitations, shows that the oral microbiome deserves to be considered as an explanatory variable in the investigation of perireceptor events involved in the perception of taste (Feng et al., 2018).

Both taste perception and smell recognition are essential determinants of food choice. Catamo et al. (2021), evaluated the taste perception and smell recognition in DM2 patients and in healthy patients. In addition, they analyzed the association of chemosensory deficiencies with anthropometric and clinical results such as Body Mass Index, fasting glycemia, medications use, cardiovascular diseases and hypertension. The study included 94 DM2 patients and 244 healthy patients. The results showed that a higher percentage of DM2 patients presented impaired taste perception of salt (22% vs. 5% in healthy patients) and impaired smell recognition (55% vs. 27% in healthy individuals). In addition, 65% of hypertensive/DM2 patients had impaired smell recognition against 18% of non-hypertensive/DM2 patients. Interestingly, patients presenting impaired taste perception and impaired smell recognition had higher fasting glycemia (149.6 mg/dL) compared with normal tasters and normal smell recognition patients (124.3 mg / dL, p-value = 0.04). These data suggest that DM seems to interfere with taste perception and smell recognition being these effects not influenced by confounding factor such as age, fasting glycemia, medications use or the presence of cardiovascular disease. Further studies are needed though to clarify this complex interplay between sensory impairment and DM and to assess the role played by the impaired taste perception and smell recognition on DM onset (Catamo et al., 2021).

* Advanced age

The oral microbial composition can be affected by several factors, including age, oral health status, dietary habits, among others. For example, in young individuals the microbial composition is more diverse compared to that of adults (Burcham et al., 2020). It has been discussed that microbial changes associated with aging might be associated with increase in the prevalence and severity of periodontitis in the elderly (Feres et al., 2016). Moreover, increasing evidence has shown that the chronic low-grade systemic inflammation that is seen during aging is an important risk factor for the morbidity and the mortality in the elderly. Yet, this chronic inflammation can be enhanced by products synthetized by the microbial communities found all over the human body, including those from gut and from subgingival biofilms, which reach the bloodstream. In view of these mechanisms, it has been claimed that treatment of periodontitis is essential, not only for improving the elderly oral health conditions, but also for improving the systemic health of the affected individuals (Moutsopoulos and Madianos, 2006; Singh and Newman, 2011; Franceschi and Campisi, 2014; Feres et al., 2016). Singh et al. (2019) analyzed 65 individuals aged 70 to 82 years who were divided into healthy aging (HA) and unhealthy aging (NHA) cohorts that differed in the occurrence of one or more of the main diseases: cancer, acute or chronic cardiovascular disease, acute or chronic lung diseases, DM, stroke or neurodegenerative diseases. The microbiome of saliva and feces were analyzed to determine microbial diversity. In contrast to the gut microbiome, where no changes were observed, the saliva microbiome showed greater alfa diversity in HA compared to the NHA group. It was also observed that the genus *Akkermansia* spp was significantly more abundant in the gut microbiota of the HA group. *Akkermansia muciniphila* is a colon mucin-degrading bacterium and it is believed that this microorganism has beneficial effects on gastrointestinal health, especially in cases of DM and obesity. Erysipelotrichaceae UCG-003 also presented a high abundance in the HA cohort. The only genus in which a significant decrease in abundance was observed in both the oral and gut microbiomes of the HA cohort compared to the NHA cohort was Streptococcus spp (Singh et al., 2019).

Islas-Granillo et al. (2019) evaluated the differences in the distribution of the various oral health indicators among elderly people (≥60 years) presenting multimorbidity, including DM. The prevalence of multimorbidity was 27.3% while the prevalence of not functional dentition was 89.9%, hyposalivation was 59.7%; edentulism was 38.9% and self-reported xerostomia was 25.2%. Dental caries was observed in 95.3% of the individuals while severe periodontitis was observed in 80%. There was a higher prevalence of edentulism in individuals with multimorbidity (55.3%) when compared to those without multimorbidity (32.7%) (Islas-Granillo et al., 2019).

* Polycystic ovary syndrome

Polycystic ovary syndrome (PCOS) is one of the most common endocrine and metabolic disorders in pre-menopausal women. As a multifactorial syndrome, PCOS is defined by a combination of signs and symptoms of androgen excess and ovarian dysfunction. The etiology of this syndrome remains largely unknown, but growing evidence suggests that PCOS may be a complex multigenic disease with epigenetic and environmental influences, including diet and lifestyle factors. PCOS is often associated with abdominal adiposity, insulin resistance, metabolic disorders and cardiovascular risk factors (Escobar-Morreale, 2018). Besides that, hyperandrogenism (excess of androgen) induces obesity, hairy, acne, and androgenetic alopecia (Zeng et al., 2020). Moreover, almost 50% of women with PCOS are obese (Messinis et al., 2015). DM2 and PCOS are commonly found as associated conditions. Whereas impaired glucose tolerance is commonly found in women with PCOS, an increased risk of developing polycystic ovaries has also been found in women with DM2. Implementation of lifestyle interventions (in terms of diet and frequent physical activity) is one of the therapies for PCOS (Pani et al., 2020). Pani et al. (2020) discussed whether the treatment of DM improves metabolic profile in PCOS women. Improving insulin sensitivity can normalize endocrine and reproductive disorders. Clinical evidence has shown how the use of insulin sensitizing agents, such as metformin and inositol, can improve the endocrine and metabolic profile in women with PCOS. However, the authors suggest that long-term controlled clinical trials using insulin-sensitizing agents in conjunction with lifestyle interventions are needed to further prove their effectiveness in reducing the incidence of DM2 and improving the metabolic and endocrine profile in women with PCOS (Pani et al., 2020).

Another common association is between PCOS and DMG. PCOS and DMG are very common conditions that increase the risk of pregnancy complications, including early pregnancy loss, pregnancy-induced hypertensive disorders and premature preterm labor, among many others. In these cases, insulin resistance plays a fundamental role in the pathogenesis of PCOS and DMG, representing an important therapeutic target for the management of both conditions. Metformin, an insulin sensitizing agent, has been shown to reduce the rates of early pregnancy loss and the onset of DMG in women with PCOS (Rojas et al., 2014).

It has already been shown in this review the interplay between oral microbiota and DM. However, data regarding the correlation between oral complications and PCOS are controversial. Akcalı et al. (2014) investigated if the levels of periodontal pathogens in saliva and their respective antibody in serum are elevated in women presenting PCOS. The studied groups were women with PCOS and healthy periodontium, women with PCOS and gingivitis, systemic health and periodontium healthy women and systemic healthy women with gingivitis. In women with PCOS, the salivary levels of *P. gingivalis*, *F. nucleatum*, *S. oralis* and *T. forsythia* were higher than in systemic healthy women. The levels of *A. actinomycetemcomitans* and *T. denticola* were similar among the studied groups. The presence of PCOS and gingivitis also increased the levels of serum antibodies against *P. gingivalis*, *P. intermedia* and *S. oralis*. In conclusion, women with PCOS tend to present a distinct oral microbial composition and an elevated systemic response to selective members of this microbial community. Akcalı et al. (2015) and Varadan et al. (2019) also showed that gingival inflammation is more likely to be found in women with PCOS compared to women without the presence of PCOS. Lindheim et al. (2016) showed lower abundance of Actinobacteria in saliva samples of PCOS women compared with healthy ones.

REFERENCES

Amou, T., Hinode, D., Yoshioka, M., Grenier, D. (2014). Relationship between halitosis and periodontal disease - associated oral bacteria in tongue coatings. Int. J. Dent. Hyg. 12, 145-151. doi: 10.1111/idh.12046.

Aylıkcı, B., and Çolak, H. (2013). Halitosis: From diagnosis to management. J. Nat. Sci. Biol. Med. 4, 14–23. doi: 10.4103/0976-9668.107255.

Burcham, Z.M., Garneau, N.L., Comstock, S.S., Tucker, R.M., Knight, R., Metcalf, J.L. (2020). Patterns of oral microbiota diversity in adults and children: a crowdsourced population study. Sci. Rep. 10: 2133. doi: 10.1038/s41598-020-59016-0.

Catamo, E., Tornese, G., Concas, M.P., Gasparini, P., Robino, A. (2021). Differences in taste and smell perception between type 2 diabetes mellitus patients and healthy controls. Nutr. Metab. Cardiovasc. Dis. 31, 193-200. doi: 10.1016/j.numecd.2020.08.025.

Choi, S.J., Jang, B.H., Lee, S.J., Min, B.K., Rothschild, A., Kim, I.D. (2014). Selective detection of acetone and hydrogen sulfide for the diagnosis of diabetes and halitosis using SnO(2) nanofibers functionalized with reduced graphene oxide nanosheets. ACS Appl. Mater. Interfaces. 6, 2588-2597. doi: 10.1021/am405088q.

Costello, B.L., Amann, A., Al-Kateb, H., Flynn, C., Filipiak, W., Khalid, T., et al. (2014). A review of the volatiles from the healthy human body. J. Breath. Res. 8:014001. doi: 10.1088/1752-7155/8/1/014001.

Dharmawardana, N., Woods, C., Watson, D.I., Yazbeck, R., Ooi, E.H. (2020). A review of breath analysis techniques in head and neck cancer. Oral Oncol. 104:104654. doi: 10.1016/j.oraloncology.2020.104654.

Feng, Y., Licandro, H., Martin, C., Septier, C., Zhao, M., Neyraud, E, et al. (2018). The associations between biochemical and microbiological variables and taste differ in whole saliva and in the film lining the tongue. Biomed. Res. 2018: 2838052. doi: 10.1155/2018/2838052.

Feres, M., Teles, F., Teles, R., Figueiredo, L.C., Faveri, M. (2016).The subgingival periodontal microbiota of the aging mouth. Periodontol 2000. 72, 30-53. doi: 10.1111/prd.12136.

Franceschi, C., and Campisi, J. (2014). Chronic inflammation (inflammaging) and its potential contribution to age-associated diseases. J. Gerontol. A. Biol. Sci. Med. Sci. 1:S4-9. doi: 10.1093/gerona/glu057.

Galassetti, P.R., Novak, B., Nemet, D., Rose-Gottron, C., Cooper, D.M., Meinardi, S., et al. (2005). Breath ethanol and acetone as indicators of serum glucose levels: an initial report. Diabetes Technol. Ther. 7, 115-123. doi: 10.1089/dia.2005.7.115.

Garcia, E., Shalaurova, I., Matyus, S.P., Oskardmay, D.N., Otvos, J.D., Dullaart, R.P.F., et al. (2020). Ketone bodies are mildly elevated in subjects with Type 2 Diabetes Mellitus and are inversely associated with insulin resistance as measured by the lipoprotein insulin resistance index. J. Clin. Med. 9: 321. doi: 10.3390/jcm9020321.

Hampelska, K., Jaworska, M.M., Babalska, Z.Ł., Karpiński, T.M. (2020). The role of oral microbiota in intra-oral halitosis. J. Clin. Med. 9: 2484. doi: 10.3390/jcm9082484.

Islas-Granillo, H., Borges-Yañez, S.A., Navarrete-Hernández, J.J., Veras-Hernández, M.A.l., Casanova-Rosado, J.F., Minaya-Sánchez, M., et al. (2019). Indicators of oral health in older adults with and without the presence of multimorbidity: a cross-sectional study. Clin. Interv. Aging. 14, 219–224. doi: 10.2147/CIA.S170470.

Laffel, L. (1999). Ketone bodies: a review of physiology, pathophysiology and application of monitoring to diabetes. Diabetes Metab. Res. Rev. 15, 412-426. doi: 10.1002/(sici)1520-7560(199911/12)15:6<412::aid-dmrr72>3.0.co;2-8.

Loesche, W.J., and Kazor, C. (2002). Microbiology and treatment of halitosis. Periodontol 2000. 28, 256-79. doi: 10.1034/j.1600-0757.2002.280111.x.

Mazzone, P.J. (2008). Analysis of volatile organic compounds in the exhaled breath for the diagnosis of lung cancer. J. Thorac. Oncol. 3, 774-80. doi: 10.1097/JTO.0b013e31817c7439.

Moutsopoulos, N.M., and Madianos, P.N. (2006). Low-grade inflammation in chronic infectious diseases: paradigm of periodontal infections. Ann. N. Y. Acad. Sci. 1088, 251-264. doi: 10.1196/annals.1366.032.

Nakano, Y., Yoshimura, M., Koga, T. (2002). Correlation between oral malodor and periodontal bacteria. Microbes Infect. 4, 679–683. doi: 10.1016/s1286-4579(02)01586-1

Nakhleh, M.K., Amal, H., Jeries, R., Broza, Y.Y., Aboud, M., Gharra, A., et al. (2017). Diagnosis and classification of 17 diseases from 1404 subjects via pattern analysis of exhaled molecules. ACS Nano. 11, 112–125. doi: 10.1021/acsnano.6b04930.

Peel, A.M., Wilkinson, M., Sinha, A., Loke, Y.K., Fowler, S.J., Wilson, A.M. (2020). Volatile organic compounds associated with diagnosis and disease characteristics in asthma - a systematic review. Respir. Med. 169:105984. doi: 10.1016/j.rmed.2020.105984.

Persson, S., Edlund, M.B., Claesson, R., Carlsson, J. (1990). The formation of hydrogen sulfide and methyl mercaptan by oral bacteria. Oral Microbiol. Immunol. 5, 195–201. doi: 10.1111/j.1399-302x.1990.tb00645.x.

Phillips, M. (1992). Breath tests in medicine. Sci. Am. 267, 74–79. doi: 10.1038/scientificamerican0792-74.

Rondanelli, M., Perdoni, F., Infantino, V., Faliva, M.A., Peroni, G., Iannello, G., et al. (2019). Volatile organic compounds as biomarkers of gastrointestinal diseases and nutritional status. J. Anal. Methods. Chem. 2019: 7247802. doi: 10.1155/2019/7247802.

Shirasu, M., Touhara, K. (2011). The scent of disease: volatile organic compounds of the human body related to disease and disorder. J. Biochem. 150, 257-266. doi: 10.1093/jb/mvr090.

Singh, H., Torralba, M.G., Moncera, K.J., DiLello, L., Petrini, J., Nelson, K.E., et al. (2019). Gastro-intestinal and oral microbiome signatures associated with healthy aging. Geroscience. 41, 907-921. doi: 10.1007/s11357-019-00098-8.

Singh, T., and Newman, A.B. (2011). Inflammatory markers in population studies of aging. Ageing Res. Rev. 10, 319-329. doi: 10.1016/j.arr.2010.11.002.

Suzuki, N., Yoneda, M., Takeshita, T., Hirofuji, T., Hanioka, T. (2019). Induction and inhibition of oral malodor. Mol. Oral Microbiol. 34, 85-96. doi: 10.1111/omi.12259.

Tangerman, A., and Winkel, E.G. (2010). Extra-oral halitosis: an overview. J. Breath. Res. 4: 017003. doi: 10.1088/1752-7155/4/1/017003.

Trefz, P., Obermeier, J., Lehbrink, R., Schubert, J.K., Miekisch, W., Fischer, D.C. (2019). Exhaled volatile substances in children suffering from type 1 diabetes mellitus: results from a cross-sectional study. Sci. Rep. 9: 15707. doi: 10.1038/s41598-019-52165-x.

Wu, J., Cannon, R.D., Ji, P., Farella, M., Mei, L. (2020). Halitosis: prevalence, risk factors, sources, measurement and treatment - a review of the literature. Aust. Dent. J. 65, 4-11. doi: 10.1111/adj.12725.

Agostini, B.A., Cericato, G.O., Silveira, E.R., Nascimento, G.G., Costa, F.S., Thomson, W.M. ,et al. (2018). How common is dry mouth? Systematic review and meta-regression analysis of prevalence estimates. Braz. Dent. J. 29, 606-618. doi: 10.1590/0103-6440201802302.

Akcalı, A., Bostanci, N., Özçaka, Ö., Öztürk-Ceyhan, B., Gümüş, P., Buduneli, N., et al. (2014). Association between polycystic ovary syndrome, oral microbiota and systemic antibody responses. PLos One. 9: e108074. doi: 10.1371/journal.pone.0108074.

Akcalı, A., Bostanci, N., Özçaka, Ö., Öztürk-Ceyhan, B., Gümüş, P., Tervahartiala, T., et al. (2015). Elevated matrix metalloproteinase-8 in saliva and serum in polycystic ovary syndrome and association with gingival inflammation. Innate Immun. 21, 619-625. doi: 10.1177/1753425915572172.

Akira, S. (2006). TLR signaling. Curr. Top. Microbiol. Immunol. 311, 1-16. doi: 10.1007/3-540-32636-7_1.

Baker, J.L., and Edlund, A. (2019). Exploiting the oral microbiome to prevent tooth decay: has evolution already provided the best tools? Front. Microbiol. 9:3323. doi: 10.3389/fmicb.2018.03323.

Basu, R., O’Quinn, D.B., Silberger, D.J., Schoeb, T.R., Fouser, L., Ouyang, W., et al. (2012). Th22 cells are an important source of IL-22 for host protection against enteropathogenic bacteria. Immunity. 37, 1061–1075. doi: 10.1016/j.immuni.2012.08.024

Belkaid, Y., and Harrison, O.J. (2017). Homeostatic immunity and the microbiota. Immunity. 46, 562-576. doi: 10.1016/j.immuni.2017.04.008.

Bostanci, N., and Belibasakis, G.N. (2018). Gingival crevicular fluid and its immune mediators in the proteomic era. Periodontol 2000. 76, 68-84. doi: 10.1111/prd.12154.

Cho, E.P., Hwang, S.J., Clovis, J.B., Lee, T.Y., Paik, D.I., Hwang, Y.S. (2012). Enhancing the quality of life in elderly women through a programme to improve the condition of salivary hypofunction. Gerodontology. 29:e972–980. doi:10.1111/j.1741-2358.2011.00594.x

Crump, K.E., and Sahingur, S.E. (2016). Microbial nucleic acid sensing in oral and systemic diseases. J. Dent. Res. 95, 17-25. doi: 10.1177/0022034515609062.

de Nardo D. (2017). Activation of the innate immune receptors: guardians of the micro galaxy: activation and functions of the innate immune receptors. Adv. Exp. Med. Biol. 1024: 1-35. doi: 10.1007/978-981-10-5987-2_1.

Escobar-Morreale, H.F. (2018). Polycystic ovary syndrome: definition, aetiology, diagnosis and treatment. Nat. Rev. Endocrinol. 14:270-284. doi: 10.1038/nrendo.2018.24.

Fábián, T.K., Hermann, P., Beck, A., Fejérdy, P., Fábián, G. (2012). Salivary defense proteins: their network and role in innate and acquired oral immunity. Int. J. Mol. Sci. 13, 4295–4320. doi: 10.3390/ijms13044295.

Fox, P.C., van der Ven, P.F., Sonies, B.C., Weiffenbach, J.M., Baum, B.J. (1985). Xerostomia: evaluation of a symptom with increasing significance. J. Am. Dent. Assoc. 110, 519-525. doi: 10.14219/jada.archive.1985.0384.

Gasner, N.S., and Schure, R.S. (2021). Periodontal Disease. In: StatPearls [Internet]. Treasure Island (FL): StatPearls Publishing. Bookshelf ID: NBK554590.

Han, S., Toker, A., Liu, Z.Q., Ohashi, P.S. (2019). Turning the Tide Against Regulatory T Cells. Front Oncol. 9: 279. doi: 10.3389/fonc.2019.00279.

Humphrey, S.P., and Williamson, R.T. (2001). A review of saliva: normal composition, flow, and function. J. Prosthet. Dent. 85, 162-169.

Hurley, E., Barrett, M.P.J., Kinirons, M., Whelton, H., Ryan, C.A., Stanton, C., et al. (2019). Comparison of the salivary and dentinal microbiome of children with severe-early childhood caries to the salivary microbiome of caries-free children. BMC Oral Health. 19:13. doi: 10.1186/s12903-018-0693-1.

Khurshid, Z., Zafar, M.S., Naseem, M., Khan, R.S., Najeeb, S. (2018). Human oral defensins antimicrobial peptides: a future promising antimicrobial drug. Curr. Pharm. 24, 1130-1137. doi: 10.2174/1381612824666180403114615.

Kim, H.J., Lee, J.Y., Lee, E.S., Jung, H.J., Ahn, H.J., Kim, B.I. (2019). Improvements in oral functions of elderly after simple oral exercise. Clin. Interv. Aging. 14, 915-924. doi: 10.2147/CIA.S205236.

Kumar, H., Kawai, T., Akira, S. (2011). Pathogen recognition by the innate immune system. Int. Rev. Immunol. 30, 16-34. doi: 10.3109/08830185.2010.529976.

Lassenius, M.I., Pietiläinen, K.H., Kaartinen, K., Pussinen, P.J., Syrjänen, J., Forsblom, C., et al. (2011). Bacterial endotoxin activity in human serum is associated with dyslipidemia, insulin resistance, obesity, and chronic inflammation. Diabetes Care. 34: 1809–1815. doi: 10.2337/dc10-2197.

Li, S., Yin, H., Zhang, K., Wang, T., Yang, Y., Liu, X., et al. (2017). Effector T helper cell populations are elevated in the bone marrow of rheumatoid arthritis patients and correlate with disease severity. Sci. Rep. 7: 4776. doi: 10.1038/s41598-017-05014-8.

Li, X., Kolltveit, K.M., Tronstad, L., Olsen, I. (2000). Systemic diseases caused by oral infection. Clin. Microbiol. Rev. 13, 547–558. doi: 10.1128/cmr.13.4.547-558.2000.

Lindheim, L., Bashir, M., Münzker, J., Trummer, C., Zachhuber, V., Pieber, T.R., et al. (2016). The salivary microbiome in Polycystic Ovary Syndrome (PCOS) and its association with disease-related parameters: a pilot study. Front. Microbiol. 7:1270. doi: 10.3389/fmicb.2016.01270.

Machiulskiene, V., Campus, G., Carvalho, J.C., Dige, I., Ekstrand, K.R., Jablonski-Momeni, A., et al. (2020). Terminology of dental caries and dental caries management: consensus report of a workshop organized by ORCA and Cariology Research Group of IADR. Caries Res. 54, 7-14. doi: 10.1159/000503309.

Marsh, P.D., and Zaura, E. (2017). Dental biofilm: ecological interactions in health and disease. J. Clin. Periodontol. 44:S12-S22. doi: 10.1111/jcpe.12679.

Messinis, I.E., Messini, C., Anifandis, G., Dafopoulos, K. (2015). Polycystic ovaries and obesity. Best Pract. Res. Clin. Obstet. Gynaecol. 29, 479-488. doi: 10.1016/j.bpobgyn.2014.11.001.

Meuric, V., Le Gall-David, S., Boyer, E., Acuña-Amador, L., Martin, B., Fong, S.B., et al. (2017). Signature of microbial dysbiosis in periodontitis. Appl. Environ. Microbiol. 83:e00462-17. doi: 10.1128/AEM.00462-17.

Moseley, T.A., Haudenschild, D.R., Rose, L., Reddi, A.H. (2003). Interleukin-17 family and IL-17 receptors. Cytokine Growth Factor Rev. 14: 155–174. doi: 10.1016/s1359-6101(03)00002-9.

Mosmann, T.R., Cherwinski, H., Bond, M.W., Giedlin, M.A., Coffman, R.L. (1986). Two types of murine helper T cell clone. I. Definition according to profiles of lymphokine activities and secreted proteins. J. Immunol. Apr 1;136(7):2348-57.

Mosmann, T.R., and Coffman, R.L. (1989). TH1 and TH2 cells: different patterns of lymphokine secretion lead to different functional properties. Annu. Rev. Immunol. 7:145-173. doi: 10.1146/annurev.iy.07.040189.001045.

Netea, M.G., Schlitzer, A., Placek, K., Joosten, L.A.B., Schultze, J.L. (2019). Innate and adaptive immune memory: an evolutionary continuum in the host's response to pathogens. Cell Host Microbe. 25:13-26. doi: 10.1016/j.chom.2018.12.006.

Pani, A., Gironi, I., Di Vieste, G., Mion, E., Bertuzzi, F., Pintaudi, B. (2020). From prediabetes to Type 2 Diabetes Mellitus in women with Polycystic Ovary Syndrome: lifestyle and pharmacological management. Int. J. Endocrinol. 2020:6276187. doi: 10.1155/2020/6276187.

Paul, W.E., and Seder, R.A. (1994). Lymphocyte responses and cytokines. Cell. 76, 241-251. doi: 10.1016/0092-8674(94)90332-8.

Pedersen, L.A.M., and Belstrom, D. (2019). The role of natural defenses in maintaining a healthy oral microbiota. J. Dent. 80:S3-S12. doi: 10.1016/j.jdent.2018.08.010.

Pina, G.M.S, Carvalho, R.M., Silva, B.S.F., Almeida, F.T. (2020). Prevalence of hyposalivation in older people: a systematic review and meta-analysis. Gerodontology. 37, 317-331. doi: 10.1111/ger.12497.

Rijkschroeff, P., Loos, B.G., Nicu, E.A. (2018). Oral polymorphonuclear neutrophil contributes to oral health. Curr. Oral Health Rep. 5, 211-220. doi: 10.1007/s40496-018-0199-6.

Rojas, J., Chávez-Castillo, M., Bermúdez, V. (2014). The role of metformin in metabolic disturbances during pregnancy: Polycystic Ovary Syndrome and Gestational Diabetes Mellitus. Int. J. Reprod. Med. 2014:797681. doi: 10.1155/2014/797681.

Romo, J.A., Pierce, C.G., Chaturvedi, A.K., Lazzell, A.L., McHardy, S.F., Saville, S.P., et al. (2017). Development of anti-virulence approaches for candidiasis via a novel series of small-molecule inhibitors of Candida albicans filamentation. mBio. 8:e01991-17. doi: 10.1128/mBio.01991-17.

Sakaguchi, S., Miyara, M., Costantino, C.M., Hafler, D.A. (2010). FOXP3+ regulatory T cells in the human immune system. Nat. Rev. Immunol. 10, 490-500. doi: 10.1038/nri2785.

Sakanaka, A., Kuboniwa, M., Hashino, E., Bamba, T., Fukusaki, E., Amano, A. (2017). Distinct signatures of dental plaque metabolic byproducts dictated by periodontal inflammatory status. Sci. Rep. 7:42818. doi: 10.1038/srep42818.

Saleh, J., Figueiredo, M.A.Z., Cherubini, K., Salum, F.G. (2015). Salivary hypofunction: an update on aetiology, diagnosis and therapeutics. Arch. Oral Biol. 60, 242-255. doi: 10.1016/j.archoralbio.

Seo, K., and Kim H-N. (2020). Effects of oral health programmes on xerostomia in community-dwelling elderly: A systematic review and meta-analysis. Int. J. Dent. Hyg. 18, 52-61. doi: 10.1111/idh.12418.

Sztukowska, M.N., Roky, M., Demuth, D.R. (2019). Peptide and non-peptide mimetics as potential therapeutics targeting oral bacteria and oral biofilms. Mol. Oral Microbiol. 34, 169-182. doi: 10.1111/omi.12267.

Takahashi, N., and Nyvad, B. The role of bacteria in the caries process ecological perspectives. (2011). J. Dent. Res. 90, 294–303.

Tan, E.C.K., Lexomboon, D., Sandborgh-Englund, G., Haasum, Y., Johnell, K. (2018). Medications that cause dry mouth as an adverse effect in older people: a systematic review and meta-analysis. J. Am. Geriatr. Soc. 66, 76-84. doi: 10.1111/jgs.15151.

Taylor, J.J., and Preshaw, P.M. (2016). Gingival crevicular fluid and saliva. Periodontol 2000. 70, 7-10. doi: 10.1111/prd.12118.

Turvey, S. E., and Broide, D.H. (2010). Innate immunity. J. Allergy Clin. Immunol. 125:S24–S32. doi: 10.1016/j.jaci.2009.07.016.

Van Dyke, T.E., Bartold, P.M., Reynolds, E.C. (2020). The nexus between periodontal inflammation and dysbiosis. Front. Immunol. 11:511. doi: 10.3389/fimmu.2020.00511. Varadan, M., Gopalkrishna, P., Bhat, P.V., Kamath, S.U., Krithishree, S., Thriveni, G.K., et al. (2019). Influence of polycystic ovary syndrome on the periodontal health of Indian women visiting a secondary health care centre. Clin. Oral Investig. 23, 3249-3255. doi: 10.1007/s00784-018-2741-2.

Villa, A., Connell, C.L., Abati, S. (2015). Diagnosis and management of xerostomia and hyposalivation. Ther. Clin. Risk Manag. 11, 45–51. doi: 10.2147/TCRM.S76282.

Xiao, J., Grier, A., Faustoferri, R.C., Alzoubi, S., Gill, A.L., Feng, C., et al. (2018). Association between oral Candida and bacteriome in children with severe ECC. J. Dent. Res. 97:1468-1476. doi: 10.1177/0022034518790941.

Zeng, X., Xie, Y-J., Liu, Y-T., Long, S-L., Mo, Z-C. (2020). Polycystic ovarian syndrome: correlation between hyperandrogenism, insulin resistance and obesity. Clin. Chim. Acta. 502:214-221. doi: 10.1016/j.cca.2019.11.003.

Zhang, Y., Wang, X., Li, H., Ni, C., Du, Z., Yan F. (2018). Human oral microbiota and its modulation for oral health. Biomed. Pharmacother. 99:883-893. doi: 10.1016/j.biopha.2018.01.146.

Zheng, D., Timur, L., Elinav E. (2020). Interaction between microbiota and immunity in health and disease. Cell Res. 30, 492–506. doi: 10.1038/s41422-020-0332-7.

Zhu, J., Paul, W.E. (2010). Heterogeneity and plasticity of T helper cells. Cell Res. 20, 4–12. doi: 10.1038/cr.2009.138.

Zhu, J. (2018). T helper cell differentiation, heterogeneity, and plasticity. Cold Spring Harb Perspect. Biol. 10: a030338. doi: 10.1101/cshperspect.a030338.
